# Predicting S_1_ TDDFT Energies from ZINDO Calculations Using Message-Passing ΔML with Electronically Informed Descriptors

**DOI:** 10.1021/acs.jctc.5c01587

**Published:** 2026-01-22

**Authors:** Adam Coxson, Ömer H. Omar, Marcos del Cueto, Alessandro Troisi

**Affiliations:** Department of Chemistry, 4591 University of Liverpool, Liverpool L69 7ZX, U.K.

## Abstract

We present a machine learning approach (ΔML) capable of enhancing the accuracy of semiempirical excited-state energy calculations to a level close to that of Time-Dependent Density Functional Theory (TDDFT). Using a data set of 7600 organic π-conjugated molecules calculated at the ZINDO and M06-2X/3-21G* TDDFT computational levels, we trained a set of models to learn the systematic errors of the low-level method and correct it toward higher-level accuracy values. The best performing model improved the correlation of ZINDO S_1_ energy predictions from 0.77 to 0.96 on a 9500 molecule test set of TDDFT target energies. Our ΔML-ZINDO model presents a negligible additional cost (∼2 ms per molecule) to a standard ZINDO calculation (∼2 s per molecule), enabling the computational screening of large data sets of molecules. Critical to the performance of the model is the AttentiveFP Message-Passing Neural Network with added electronic information derived from ZINDO calculations such as particle-hole densities. We also investigate the utility of the Morgan fingerprint and a novel descriptor designed to capture the electronic structure of molecules: a molecular orbital-weighted radial distribution function. The ΔML framework is retrainable to other low- and high-level calculation pairs, achieving an improvement in correlation from 0.88 to 0.99 on a test set of 24,000 molecules from the QCDGE data set, when mapping ZINDO to ωB97X-D/6-31G* energies. We also adapt ΔML-ZINDO for S_1_ oscillator strength prediction, improving ZINDO predictions from a correlation of 0.524 to 0.839 on our M06-2X/3-21G* target test set, thus enabling the identification of emissive molecules.

## Graphical abstract

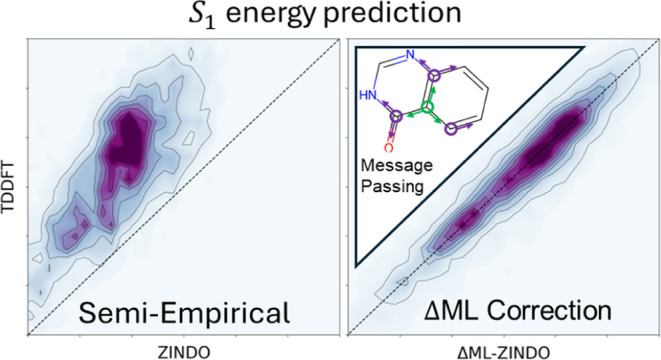

## 1 Introduction

Machine Learning (ML) has become an integral tool in many areas of computational chemistry^1,2^ and offers enormous potential to enhance both the accuracy and speed of standard quantum chemistry. Popular applications of ML include the acceleration of molecular dynamics,^3^ density functional approximations,^4^ and the development of interatomic potentials and force fields.^5,6^ One desirable and intuitive application is to use ML to account for systematic errors in approximative physics-based models and improve their accuracy at only a marginal additional cost. Known as Δ-learning, or ΔML, these methods cross-examine low- and high-level quantum chemical calculations to build data-driven models that correct the errors of the least accurate method.^7^ ML methods work well in high-throughput virtual screening (HTVS)^8,9^ and particularly excel when trained on large and representative data sets, which can be built from open-source chemical databases, such as PubChem^10^ and ZINC.^11^

For any given simulation problem, there is a multitude of quantum chemical methods available, but the most accurate methods come at a considerably greater computational cost, which makes them unsuitable for HTVS applications. Usually, the majority of the underlying physics of a molecular system is already captured by efficient low-level methods, while the high-level methods achieve greater accuracy by accounting for subtle physical effects, such as higher-order electron correlations^12^ or long-range dispersion interactions,^13^ which come at a disproportionately higher cost. ΔML methods can be used to predict (and therefore correct) the systematic errors present in the low-level theory by analyzing data sets that contain properties computed at both the low- and high-level methods.^14^ For example, in 2015, a generalized ΔML model based on kernel ridge regression (KRR) was trained on 13,000 organic molecules for a range of low-level semiempirical and DFT methods, which were corrected up to the greater chemical accuracy of the higher-level G4MP2 target.^7,15^ This approach reduced the error of semiempirical baselines from 7.2 kcal/mol to the DFT standard of 3 kcal/mol at the computational cost of the semiempirical method. Later in 2020, Dral et al. further developed the approach to show how a hierarchical ΔML model utilizing an ensemble of baseline–target combinations enabled the modeling of potential energy surfaces at *ab initio* accuracies, while reducing the computational cost by a factor of 100.^16^ In other applications, Collins and Raghavachari predicted the thermochemistry of large molecules by using ΔML to combine molecular fragments,^17^ and Ward et al. demonstrated how ΔML could consistently outperform standard ML and transfer-learning approaches for both kernel-based and network-based methods.^18^ Chen et al.^19^ used ΔML to predict solution-phase molecular properties by evaluating a range of descriptors covering solvent–solute features, energy components, the Coulomb matrix, and fingerprints that combined the Morgan fingerprint with solvent descriptor terms.

Message-passing neural networks (MPNNs) have proven to be well suited for ΔML applications. For example, Verma et al.^20^ used MPNNs to correct xTB-sTDA first excited-state energies onto B310 to 25 TDDFT targets, which they compared to Direct-ML approaches that only rely on the molecular structure descriptors without using the xTB-sTDA energy. For the 300 k molecule MOPSSAM training set, the xTB-ΔML, Direct-ML, and xTB-sTDA models achieved MAEs of 0.11, 0.14, and 0.24 eV, respectively. Another MPNN-based ΔML model accounted for the inclusion of perturbative triples by learning the deviations between CCSD and CCSD­(T) level predictions.^21^ Taking single- and multitask models into account, Kenneth et al.^22^ used E(3)-invariant MPNNs on GFN2-xTB calculations to predict a range of properties at the wB97X-D/def2-SVP level, such as the HOMO–LUMO gap, where ΔML achieved a mean absolute error (MAE) of 0.047 eV compared to 0.053 eV for the Direct-ML model. Despite the versatility of the approach, many ΔML efforts have only trained on small molecule data sets, like QM9,^23^ that have limited diversity^24^ and do not exhibit the phenomena found in larger systems, such as extended π-conjugation and nonlocality. Furthermore, while there are studies that focus on excited-state modeling,^19,20,25–27^ they do not consider how to specifically optimize the diversity of the electronic structure when sampling their training data sets. Finally, ΔML approaches typically fail to exploit the full electronic information provided by low-level methods: The OrbitAll^28^ model set itself apart from others by using all available information from spin-enhanced GFN1-xTB calculations to predict B3LYP-D3­(BJ)/6-311+G­(d,p) target energies. OrbitAll beat a range of other ML models, sometimes only requiring 10% of the training data and offering a 1000× speed up over standard B3LYP-D3­(BJ)/6-311+G­(d,p) calculations.^28^ Some other examples of ΔML can be found in refs 27,29–33 .

To model the excited states of organic molecules, many studies have evaluated the accuracy of TDDFT calculations^34–39^ exploring between several thousands and several tens of thousands of molecules^26,40–42^ and showing that TDDFT results are in excellent agreement with experimental values.^43,44^ Achieving the fast evaluation of excited states will enable HTVS efforts^8,45,46^ to identify novel molecules with specific optical properties (e.g., organic light-emitting diodes,^47^ photovoltaic devices^48^). However, many databases contain hundreds of millions of molecules for which TDDFT screening methods are intractable.

The goal of this work is to develop a ΔML approach that can achieve TDDFT-level S_1_ energy predictions at the cost of a ZINDO^49^ calculation for future integration into rapid molecular screening workflows. It will train a message-passing neural network on a data set of extended π-conjugated organic molecules with 10 to 25 heavy atoms, using structural- and electronic-type descriptors derived from ZINDO calculations, thus capitalizing on the rich variety of excitation data offered by ZINDO. TDDFT with the M06-2X functional^50^ was chosen as the target reference for $E^{S_{1}}$ values, as it shows an excellent correlation of *r* ≃ 0.95 with experiment, as per an analysis by Wu et al.,^51^ see Section C in the Supporting Information (SI) for more details. The semiempirical ZINDO is a suitable low-level input method as it is up to 1000 times faster^52^ and produces excited-state energies that are reasonably well correlated with TDDFT at *r* ≃ 0.83 (see Figure 1).

**Figure 1:**
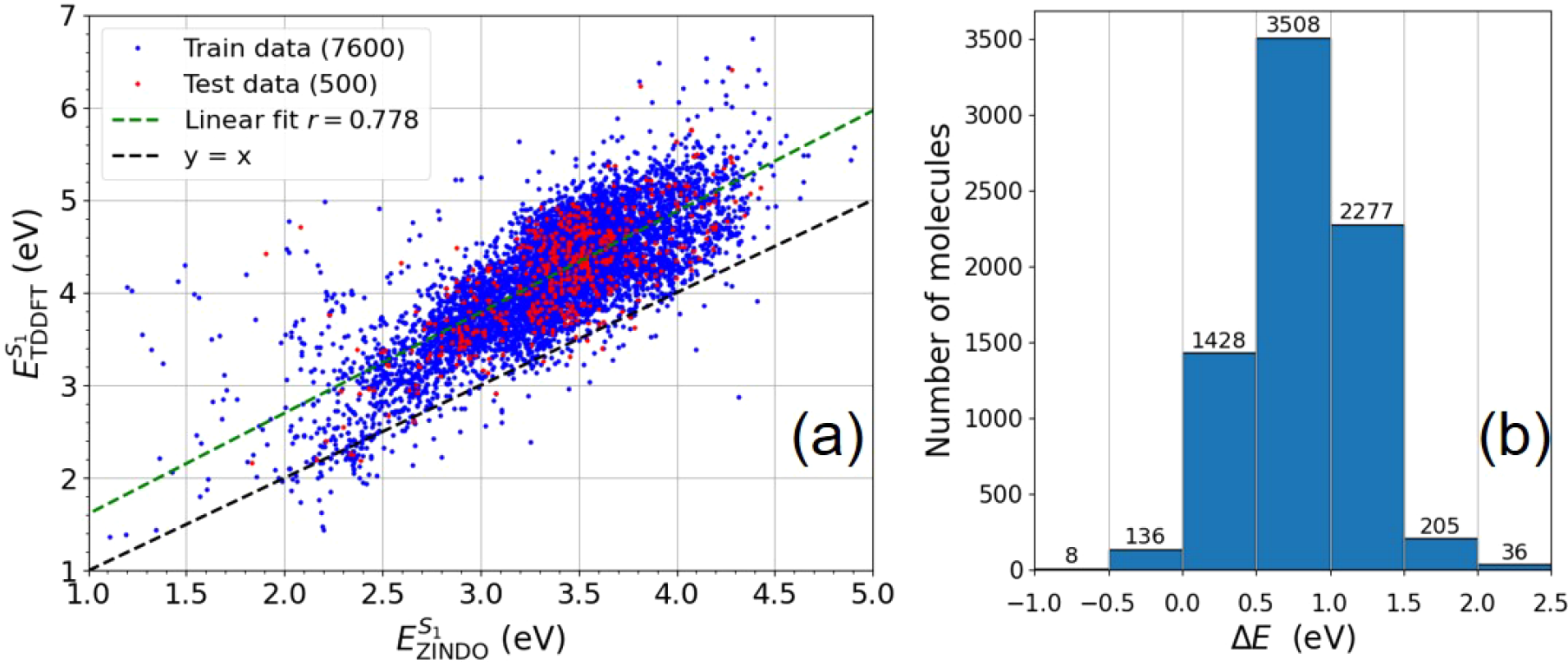
True distribution of the ZINDO and TDDFT first excited-state energies (a) and their differences (b). In (a), we see training data points in blue and a random sample of 500 test points in red, for which the linear model obtained *r*_train_ = 0.778 and *r*_test_ = 0.769.

While ΔML frameworks are already well established, the three novel contributions of this work are as follows: (1) The formation of a unique-core ZINC-derived data set optimized for electronic structural diversity, as described in Section 2. (2) The modification of the AttentiveFP Graph Neural Network to include electronically informed atomwise terms as nodal information, which we evaluate in Section 5.1. (3) The introduction of a novel Molecular Orbital Radial Distribution Function descriptor, which we evaluate in Section 5.2. These contributions were designed specifically to introduce electronic information to the learning algorithms, going beyond purely geometry-based descriptors commonly found in molecular machine learning literature.^25,53,54^ To further illustrate the generalizability of our framework, in Section 5.4 we apply it to the QCDGE data set^55^ and adapt our model to predict S_1_ oscillator strengths. We provide the train-test data set, pretrained models, and training tutorials to reproduce the work in a public GitHub repository.^56^

## 2 Data Set

The availability of high-quality excited-states data sets is limited; there are only 3 notable examples:

1 The high-volume PubChemQC database^57^ has a subset of 2 million molecules for which data on the first 10 low-lying excited states are available, with both geometries and single points at the B3LYP/6-31+G* TDDFT level.
2 The QM-symex data set^41^ contains 173k molecules of up to 60 heavy atoms, comprised of H, B, C, N, O, F, Cl, and Br, with geometries and TDDFT single points for the first 10 states calculated at the B3LYP/6-31G* level.
3 The QCDGE data set^55^ aggregates molecules from the QM9,^23^ GDB-11,^58^ and PubChemQC^57^ databases to form a data set of 443k small organic molecules with up to 10 CNOF heavy atoms. Geometries are available at the B3LYP/6-31G*-BJD3 level, with ωB97X-D/6-31G* TDDFT single points.

Data set suitability for machine-learning electronic structure depends on factors such as volume, level of theory, chemical diversity, and underlying molecule generation/sampling methodology. As noted by Glavatskikh et al. (PC9 data set),^24^ QM9/GDB-derived data sets can exhibit limited diversity due to enumeration-based generation; additional constraints such as low heavy-atom counts or application-specific filtering (e.g., QM-symex’s emphasis on molecular symmetry groups) further restrict coverage. Much larger data sets do exist, but they often provide excited-state information at more approximate levels of theory, such as the ORNL-AISD data set^59^ of 10.5 M molecules of up to 71 CNOSF heavy atoms with TD-DFTB excited states. To date, we are not aware of data sets that simultaneously exceed 200k molecules, extend to more than 10 heavy atoms, and go significantly beyond the TDDFT/6-31G* level.

Considering these points, we introduce a new excited-state data set designed for high-throughput screening workflows in organic electronics. It matches contemporary data sets in the level of theory while substantially increasing data set volume and *electronic* diversity. We started with 12 M commercially available molecules from the (2015) ZINC database^11^ and applied a common-core clustering approach to obtain a subset of 150,000 molecules each with a unique conjugated core, i.e., all differing in the network of π-conjugated bonding, which dominates their electronic structure and hence their S_1_ energy (and other properties). This approach, described in more detail in Section A.1 of the SI, maximizes the electronic diversity of the data set and avoids the presence of multiple molecules with almost identical electronic properties (e.g., naphthalene and 1-methyl-naphthalene). This is necessary as structural chemical diversity does not guarantee that the data set has the same degree of electronic diversity (for comparison, applying this clustering to the 10.5 million molecules in the ORNL-AISO data set reduces it to 0.5 million unique-core molecules, a 20-fold reduction).

Although the full data set comprises 150,000 unique cores, to facilitate efficient model development and prototyping, we restricted the training data to a representative subset to ensure computational tractability. We obtained 9050 candidates by selecting molecules with 10 to 25 C, N, O, S, or F heavy atoms. Furthermore, we identified that compounds containing the nitroso (−N=O) group consistently produced S_1_ energies centered around 3.3 eV, which artificially inflated the model’s prediction accuracy by up to 10% due to the ease of predicting the energy of these molecules. To ensure the data set reflected true chemical diversity rather than exploiting systematic artifacts, we removed these compounds, resulting in a final training set of 7602 unique-core molecules.

Each molecule underwent a conformational search using the UFF potential^60^ with the default OpenBabel conformer search algorithm.^61^ The 10 lowest-energy UFF conformers were then optimized with PM7,^62^ and the lowest-energy PM7 conformer was used as the common geometry for both the low-level ZINDO calculation and the subsequent DFT/TDDFT calculations (i.e., ZINDO and TDDFT are evaluated on the same PM7 conformer). For the low-level ΔML input, a single-point ZINDO calculation was applied to the PM7 geometry (ZINDO//PM7) to obtain the S_1_ excited-state data. To form the high-level target data, the PM7 geometry of each structure was further optimized at the BLYP35/3-21G* level, and then a single-point TDDFT calculation was performed using M06-2X/3-21G*, in accordance with a previously benchmarked protocol.^43^ As discussed in Section C of the SI, S_1_ energies computed with def2-TZVP and 3-21G* show an excellent correlation of 0.97, justifying the modest basis-set compromise in exchange for roughly a 90% reduction in CPU time, which makes HTVS tractable and enables a larger training set. All quantum-chemical calculations were performed using Gaussian 16.^63^

We define the term *core-analogues* to describe two different molecules with the same conjugated core but different overall chemical structures, differing by terminal functional group but not by extensions of their conjugated parts. For the unique cores in the training set, we can identify the number of analogues each training core has in the original set of 12 million molecules. We built the test set by sampling up to a maximum of 3 analogues per training set molecule, which gave 9500 extended π-conjugated molecules of up to 35 heavy atoms. This meant that for each molecule in the test set, there was exactly one molecule in the training set that had the same conjugated core. This train-test, one-to-many mapping is illustrated in the SI in Figure S3.

Figure 1a shows the correlation of a linear fit between ZINDO S_1_ energies $\left(E_{\mathrm{ZINDO}}^{S_{1}}\right)$ and TDDFT S_1_ energies $\left(E_{\mathrm{TDDFT}}^{S_{1}}\right)$ on the training data:

1 $$E_{\mathrm{Linear}}^{S_{1}}=mE_{\mathrm{ZINDO}}^{S_{1}}+c$$

From this fit, the linear model had *m* = 1.08, *c* = 0.52, and a Pearson correlation of *r*_train_ = 0.778, correcting the 0.5 eV discrepancy between ZINDO and TDDFT calculations. Applying the linear model to the test data gave *r*_test_ = 0.769, where a random selection of 500 test molecules is shown on the plot in red for clarity. The residual errors between TDDFT and ZINDO values are defined as

2 $$\Delta E=E_{\mathrm{TDDFT}}^{S_{1}}-E_{\mathrm{ZINDO}}^{S_{1}}$$

and are shown in Figure 1b, where 95% of residuals are between 0 to 1.5 eV, further highlighting the systematic underestimation of TDDFT energies by ZINDO. This fitting can be improved if molecule-specific deviations are further addressed by machine learning corrections.

As per standard practice, we performed *k*-fold cross-validation on the training data to evaluate the performance of a given architecture and its hyperparameters on the validation folds. After the selection of a suitable architecture, each fold model was applied separately to the test set.

The organization of our data set into these validation and test sets based on conjugated cores is a distinct feature of this work, designed to assess specific generalization modes. Within each *k*-fold iteration, the validation data contains unseen π-conjugated cores, effectively testing the model’s generalizability toward out-of-domain data (extrapolation). In contrast, the testing set contains core-analogues of the training molecules and evaluates in-domain electronic structures (interpolation). We report metrics for both to demonstrate performance under both extrapolative and interpolative conditions. Sections A.2 and A.3 of the SI provide further validation of the validation and training sets.

It is important to clarify that while molecules with the same conjugated core share a similar electronic structure, they do not necessarily share a similar geometry; they often differ in terminating groups. Therefore, the test set specifically evaluates the model’s ability to interpolate electronic properties across significant structural variations of known conjugated cores (i.e., common cores seen during training). One could alternatively describe our test set as *electronically interpolative but structurally extrapolative*; our training set is limited to molecules of up to 25 heavy atoms, whereas the test set contains significantly larger molecules of up to 35 heavy atoms. In this context, the aim of our approach is to learn the electronic structures of a clustered subset of unique cores to successfully extrapolate to the wider chemical space of the full data set. Furthermore, in our preprint^64^ (the early version of this work), we used a test set of 407 molecules, and as shown later in Section 5, expanding to 9500 had a negligible effect upon our conclusions, which is strong evidence of the generalizability of the model. Section A.4 of the SI provides similarity metric analysis of our ZINC-derived data set.

## 3 Descriptors

The choice of molecular descriptors is critical for any ML-based approach, and an ideal set of descriptors encodes all relevant information about a molecule. In this work, we experimented with different combinations of architectures and descriptors to find the best performing variants, while developing an understanding of which descriptor combinations encode the greatest amount of molecular information. Three types of descriptors were investigated in this work: Fingerprints of molecular structure, ZINDO-derived electronic atom-level properties (e.g., effective charges), and a novel descriptor that weights a standard radial distribution function (RDF) by molecular orbital (MO) coefficients to combine structural and electronic information.

Among the descriptors, we also add the ZINDO S_1_ energy, $E_{\mathrm{ZINDO}}^{S_{1}}$ , which is known as the Crude Estimator of the Property (CEP) as it is the low-level equivalent of the target $E_{\mathrm{TDDFT}}^{S_{1}}$ energy. CEPs increase prediction accuracy,^20^ are critical for small chemical training sets,^65^ and score highly on feature importance tests.^19,66^ Using $E_{\mathrm{ZINDO}}^{S_{1}}$ as another descriptor also makes intuitive sense in our specific application, since the S_1_ energies already share a good correlation, as shown in Figure 1. Consequently, it is important to embed this information into the data-driven latent space, as it greatly aids in the learning process.

### 3.1 Fingerprints: MorganFP and AttentiveFP

A common approach used in cheminformatics is to encode molecular functional groups into a binary array to generate a so-called “fingerprint”.^67^ This approach is meaningful as it is possible that ZINDO errors are due to the presence of specific functional groups and fingerprints are a well-established method for representing a variety of molecular fragments within a structure and for integration into property-structure models.^68,69^ In this work, we have used Morgan fingerprints (MFP) with a 2048 bit length and a 2 Å radius, as per default settings and in accordance with previous work.^70,71^ This ensures the MFP has a sufficiently large feature space, reduces bit collision, and captures the local environment up to 4 bonds without overfragmenting the fingerprint.^72^

We also create data-driven fingerprints using the AttentiveFP MPNN model, which operates on basic one-hot encodings of nuclear charges, bond hybridization, and other connectivity features as described in ref 73. Evidently, MPNNs are fully fledged stand-alone ML algorithms and are not just descriptors. However, they build latent embeddings of molecular structures, which can be either directly mapped onto target properties or used as neural fingerprints and combined with other descriptors for further embedding in later learning layers. In this work, the stand-alone AttentiveFP model will be used, as well as the neural fingerprint^74^ obtained from the AttentiveFP embedding, which is passed as input into a set of dense layers, as described in Section 4.3.

### ZINDO-Derived Charge-Based Properties: μ_*i*_, **ϕ_*i*_**, $n_{i}^{\mathrm{elec}}$ , $n_{i}^{\mathrm{hole}}$

3.2

A ZINDO calculation produces not just the $E_{\mathrm{ZINDO}}^{S_{1}}$ but also a full estimate for the electronic structure of a molecule, and as such there is a large amount of low-level data that can be exploited as extra descriptor terms, such as the orbital energy eigenvalues, density matrices,^75^ and dipole moments.^76^ To make further use of the ZINDO calculation data other than the S_1_ energy, some simple, atomwise electronic properties were extracted for use as extra nodal features in the message-passing networks. This includes the Mulliken partial charge, the atomic potentials, and S_1_ electron–hole atomic densities, μ_*i*_, ϕ_*i*_, $n_{i}^{\mathrm{elec}}$ , and $n_{i}^{\mathrm{hole}}$ , respectively.

- For each atom *i* in a given molecule, Mulliken partial charges μ_*i*_ are derived from the ZINDO one-electron reduced density matrix.^77^ These are approximate effective charges that contain a significant amount of information on the local electronic structure.^78^
- Atomic potentials are calculated from atomwise nuclear charges *Z*_*i*_ and pairwise separations *r*_*ij*_. For a molecule of *N* atoms, the atomic potential on atom *i* is given by
3 $$\phi_{i}=\overset{N-1}{\underset{j}{\sum }}\frac{Z_{j}}{r_{ij}},,\mathrm{where}\;i\notin j$$
  which is the cumulative effect of all (point) charges in the molecule at the *i*th position, weighted by their separation from *i*. Note that using *Z* means this descriptor is not strictly from the ZINDO calculation but is still related to the external potential of the electronic Hamiltonian. The Mulliken charges and atomic potential-based terms are good descriptors of the electronic structure and have been useful features in machine learning endeavors.^79–81^ This is illustrated by linking these variables to the Fock matrix, as shown in Section B.1, Figure S6, in the SI.
- Particle-hole densities are derived from a Mulliken-like population analysis of the natural transition orbitals (NTOs), which come from the transition density matrix.^82^ They are a compact representation of how different parts of the molecule contribute to electronic excitations. NTOs consist of one occupied “hole” orbital and one unoccupied “particle” orbital, which is an effective way to condense delocalized multiorbital excitations into a single electron–hole pair.^83^ As such, these particle-hole densities are excellent descriptors of electronic excitations,^84^ which we compute for the transition of interest (S_0_ → S_1_) by performing a wave function analysis^85^ on top of a ZINDO calculation to obtain the NTOs. These are summed per atom to obtain electron and hole densities $\left(n_{i}^{\mathrm{elec}},n_{i}^{\mathrm{hole}}\right)$ , which represent the *i*th atom’s contribution to the S_0_ → S_1_ transition.

### 3.3 Molecular Orbital Weighted Radial Distribution Function (MO-RDF)

Fingerprints are primarily structure- and composition-based descriptors and do not specifically encode any electronic information other than what may be inferred from nuclear charges and their positions. Molecules may have similar geometries and nearly identical Morgan fingerprints, but the nonlocality of the electronic density can result in very different electronic structures and hence different S_1_ excitation energies. It is therefore desirable to include a descriptor that contains information on the electronic structure to maximize the predictive performance of our ΔML approach. To create such a novel electronic descriptor, we have considered the shape of the highest occupied molecular orbital (HOMO) of each molecule, which is expected to determine the electronic behavior of a given molecule. We encode this using radial distribution functions (RDF)^86^ weighted by the one-electron HOMO density, which are invariant with respect to molecular orientation, a so-called Molecular Orbital RDF (MO-RDF).

Indicating with *w*_*i*_ the population of the HOMO orbital on atom *i* at position *r*_*i*_, we define the MO-RDF, *f*(*r*), of a molecule as

4 $$f\left(r\right)=\underset{i>j}{\sum }w_{i}w_{j}\cdot g\left(r-|r_{i}-r_{j}|\right)$$

5 $$g\left(x\right)=\frac{1}{\sigma \sqrt{2\pi }}e^{-x^{2}/2\sigma^{2}}$$

where *g* is a normalized Gaussian function and σ is a constant set to 0.05 Å for this work. The RDF was calculated within the range of 0.5 to 15 Å, at intervals of 0.02 Å, giving a feature vector of length 726. The RDF can be similarly weighted for any MO of choice, like the LUMO, and can also be weighted by the NTO particle densities. As illustrated in the inset of Figure 2a, we see two molecules with similar structures that produce near-identical Morgan fingerprints yet have different distributions of the HOMO, which is reflected in the MO-RDF. Similarly, as shown in Figure 2b, there are molecules with different structures and very different Morgan fingerprints, but near-identical electronic properties, where in the inset we see that the frontier orbitals are localized on similar fragments of the two molecules, and the MO-RDFs are very similar.

**Figure 2:**
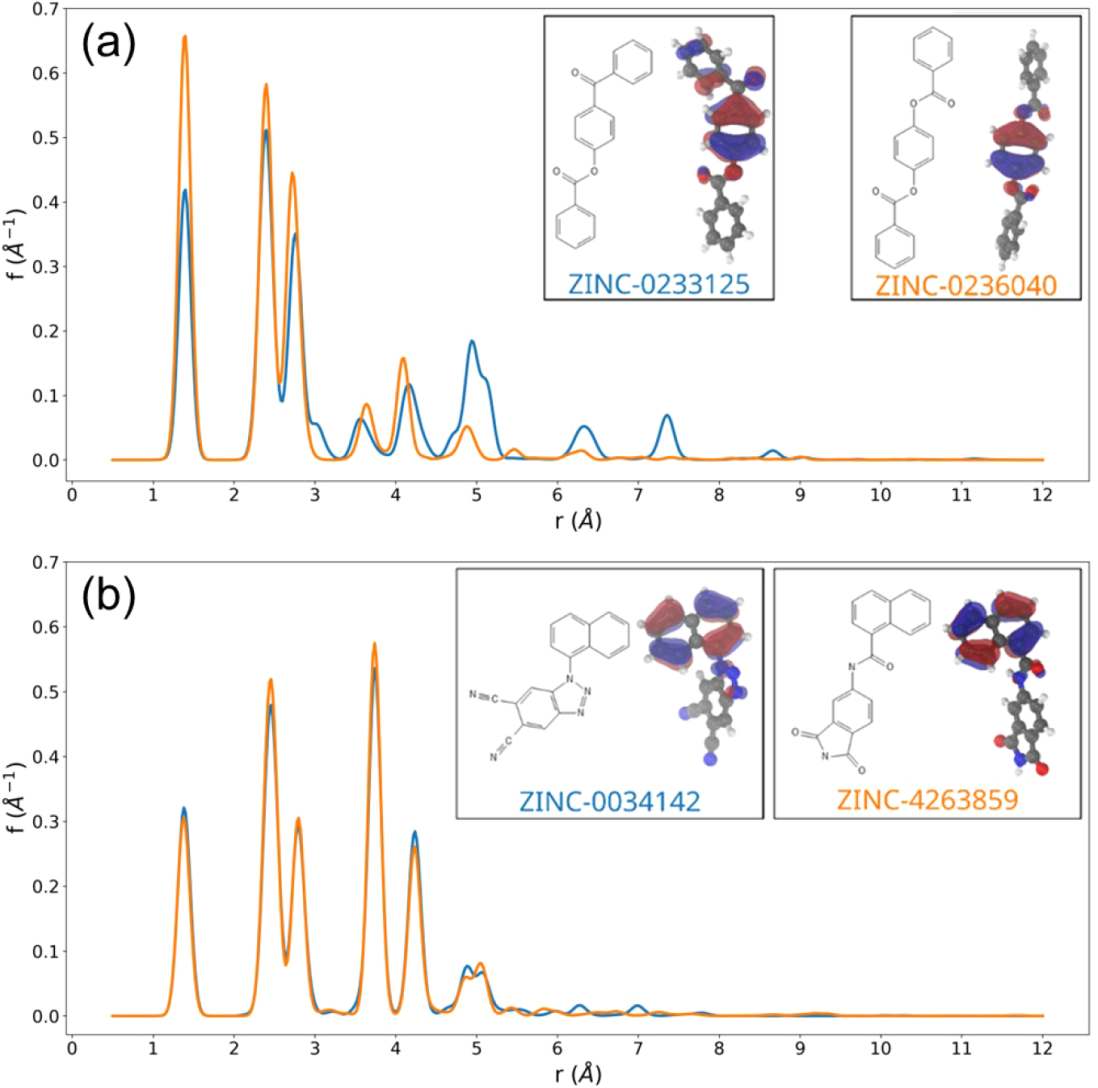
(a) MO-weighted radial distribution functions (RDFs) of two molecules whose chemical structures are almost identical, but their HOMOs differ significantly. (b) MO-weighted RDFs of two molecules whose chemical structures differ significantly, but their HOMOs are almost identical.

## 4 ML Models

### 4.1 Dense Networks

In this work to predict the electronic properties of molecules, we have considered two main architectural components: a dense neural network (also known as multilayer perceptrons/feed-forward networks)^87^ and the AttentiveFP message-passing neural network (MPNN).^73^ Dense networks are the most basic algorithm within machine learning but are simple to implement and intuitive to understand. They are a good starting point for a machine learning project to determine the utility of the descriptors and workflow before implementing more advanced models. Dense layers are still utilized across more advanced architectures, sometimes as interim embeddings but almost always as final layers before target prediction. Furthermore, advanced message-passing or transformer models can all be essentially reduced down to complex configurations of multilayer perceptrons.^88–91^

The models are implemented using PyTorch-based^92^ classes that allow dynamic assignment of the layers to build a dense block by adding nonlinear activation functions,^87^ dropout,^93^ and batch normalization^94,95^ where appropriate. Residual connections^96,97^ are used to propagate information from the descriptor set *x* to the final prediction *y*_pred_, which means the ML model only has to learn the deviation between the input and target, i.e., the residual Δ,

6 $$y_{\mathrm{pred}}=\mathrm{ML}\left(x\right)+x=\Delta +x$$

In our case, we only apply a residual connection to the $E_{\mathrm{ZINDO}}^{S_{1}}$ value, which is in line with the main principle of ΔML by modeling the deviation in S_1_ energies,

7 $$E_{\mathrm{TDDFT}}^{\mathrm{pred}}=\mathrm{ML}\left(x\right)+x=\Delta E^{\mathrm{pred}}+E_{\mathrm{ZINDO}}^{\mathrm{true}}$$

A typical example of the blocks that constitute the layers of a dense network is shown in Figure 3c. These dense blocks can operate on a single descriptor or set of descriptors, which in this work corresponds to groupings of the AttentiveFP embedding, MorganFP, MO-RDF, and *E*_ZINDO_, where only *E*_ZINDO_ is propagated via residual connections. A formulaic and low-level description of dense networks is available in the SI, Section B.2.

**Figure 3:**
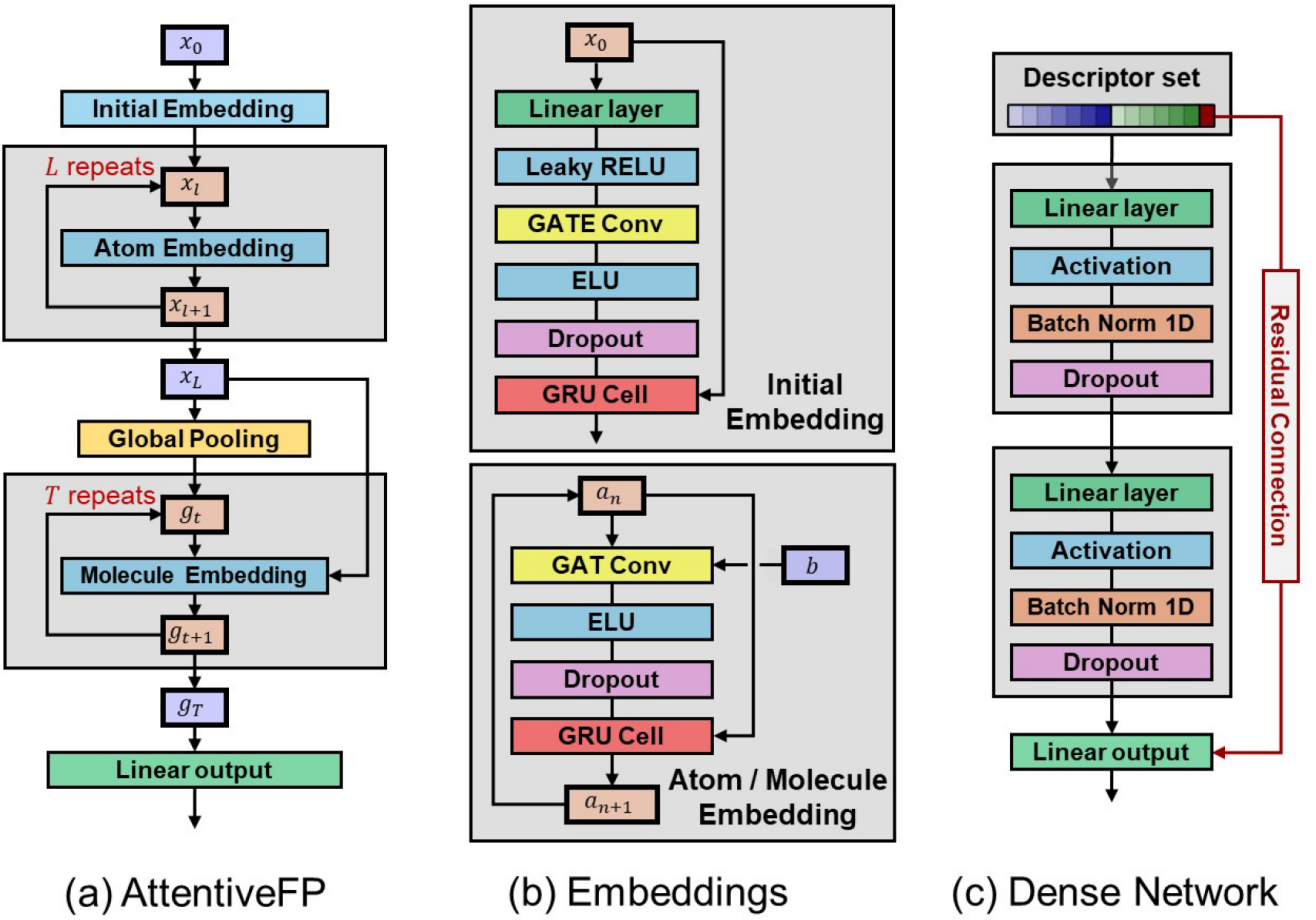
Block diagrams of layers within the ML models: (a) the flow of data through the AttentiveFP model to create a neural fingerprint (i.e., the linear output layer); (b) the layers of components within the AttentiveFP algorithm, the initial, atomic, and molecular embedding block; and (c) a 2-layer dense network with a residual skip connection.

### 4.2 Message-Passing AttentiveFP

Message-passing neural networks have proven to be exceptionally good at modeling graph based information, making them naturally well suited to molecular geometries.^98–100^ In this work, AttentiveFP was chosen as the MPNN of choice as it utilizes Graph Attention Networks (GAT),^101^ is easy to implement via the PyTorch-Geometric package, and despite the advent of many new models since AttentiveFP (2019), is still highly competitive on recent benchmarks for molecular modeling.^102–107^

The AttentiveFP MPNN model^73^ can process simple connectivity data derived from SMILES^108^ strings to build up molecular representations from atomic- and molecular-level information. The default AttentiveFP descriptors are simple one-hot encodings of graph node information (e.g., the atomic type) and edge connectivity information (e.g., the bond order). It can embed local atomic environments by propagating this information between nearest-neighbor nodes and also uses a graph attention mechanism that can adjust the embedding for nonlocal effects across the molecule. Subsequent iterations of the “message-passing” stage recursively include information from more and more distant nodes. The default AttentiveFP architecture is illustrated in Figure 3a which shows the initial embedding block (*x*_0_), the atom embedding block (*a* = *x*, *b* = None), and the molecule-level embedding (*a* = *g*, *b* = *x*_*T*_), where the only difference between the atom/molecular embedding is that the atom-level does not use the fixed *b* parameter. The main message-passing and self-attention operations are performed by GATs^101^ and GRU Cells^109,110^ as per the PyTorch Geometric implementation.^111^ The molecular-level embedding is then aggregated into a final linear output layer, which can be fitted to prediction targets or used as a deep-learning neural fingerprint for further embedding in dense networks. In this work, both forms have been used, where, in the latter case, the neural fingerprint is trained at the same time and fitted to TDDFT energies along with the rest of the hierarchical model.

### 4.3 Model Variants

In this work, the performance of the stand-alone AttentiveFP was assessed with and without the extra ZINDO-derived electronic atom descriptor terms: Mulliken charges, atomic potentials, and hole/electron particle densities, as shown in Figure 4a. The AttentiveFP default feature vector has a length of 41 input channels per atom, which was increased to 45 channels to accept the 4 additional electronic terms. Next the AttentiveFP was employed as a neural fingerprint to be evaluated with other descriptor groupings via a second stage of dense-embedding layers (Figure 4b). Many different combinations of the ML algorithms and their descriptors were tested, with illustrations of three variants shown in Figure 4. An analysis of non-MPNN-based models that only use dense blocks to process combinations of the MO-RDF, MorganFP, and ZINDO energy (Figure 4c) was also performed and can be found in the SI, in Section B.3. By evaluating the prediction accuracy of each variant, we can gain some indication of descriptor fidelity and how appropriate each one is for modeling excited states (i.e., an ablation study).

**Figure 4:**
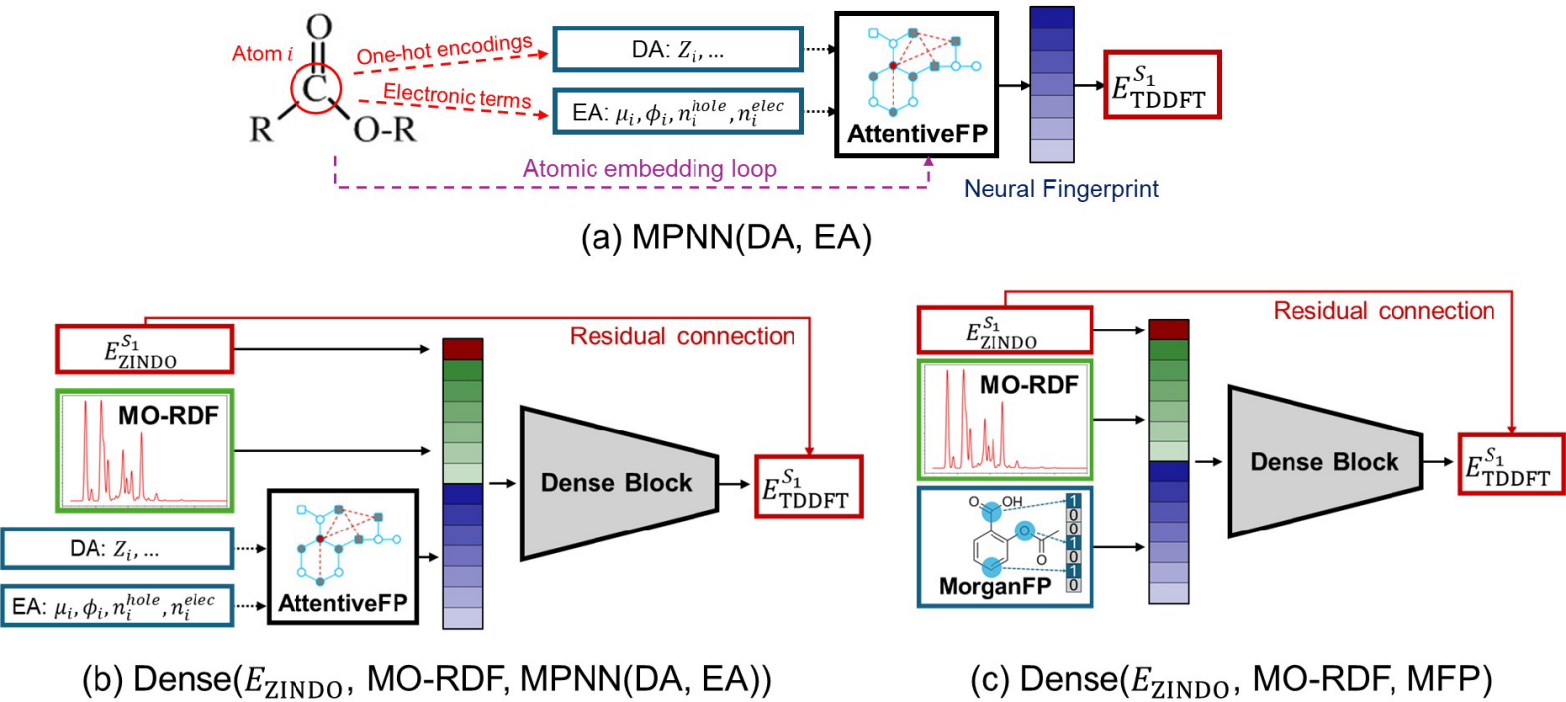
Illustrations of three main model types. (a) The stand-alone AttentiveFP model with both default atom (DA) and additional electronic atom (EA) descriptors. (b) An MPNN-based model variant that operates on the ZINDO energy, MO-RDF, and AttentiveFP neural fingerprint, which are concatenated and passed into a final dense block for TDDFT energy prediction. (c) The non-MPNN variant with the Morgan fingerprint instead of the AttentiveFP embedding. Note that not all of the different variants are shown.

To denote different ML components and descriptors, the form “component­(descriptor)” will be used throughout. When using the AttentiveFP as per ref 73, with its default atom (DA) descriptors, we will use the shorthand MPNN­(DA). When including our additional μ_*i*_, ϕ_*i*_, $n_{i}^{\mathrm{elec}}$ , and $n_{i}^{\mathrm{hole}}$ ZINDO-derived electronic atom (EA) descriptors, we will denote it MPNN­(DA, EA). To denote a dense-only network operating on the feature vector concatenation of $E_{\mathrm{ZINDO}}^{S_{1}}$ and the Morgan fingerprint, we use Dense­(*E*_ZINDO_, MFP), where we drop the S_1_ superscript for brevity. In the case of using the AttentiveFP neural fingerprint with additional electronic descriptors and further processing it with *E*_ZINDO_ and the Morgan fingerprint via dense block embeddings, we denote it as Dense­(MPNN­(DA, EA), *E*_ZINDO_, MFP). This shorthand is important to highlight the hierarchical nature of the embeddings, i.e., the combination of the neural fingerprint with other descriptors for prediction using a final dense block before back propagation throughout the entire model variant.

## 5 Results

To evaluate each model, *k*-fold cross-validation was used to train 10 different models, each with a different seeding of network weight initialization. As such, each training and validation fold was made up of approximately 6750 and 750 molecules, respectively. The performance of each fold model on its validation fold was averaged together to obtain a correlation and error estimate that is representative of the full model's uncertainty range; by randomizing both the data splits and the weight initialization, we sample different regions of the loss landscape, thereby capturing a broader spectrum of gradient-descent trajectories and increasing the upper uncertainty bound. The 10 fitted fold models were each applied to the testing set of 9500 core-analogues so their predictions could be averaged.

Initial hyperparameter optimization was performed using *k*-fold cross-validation results to determine the most appropriate settings for the activation function, learning rate, batch size, neuron configuration, dropout percentage, and whether to apply batch normalization or residual connections, for each of the different model variants. A good set of hyperparameters was identified with the Adam optimizer, 3-layer dense embeddings of 512, 256, and 32 neurons, a learning rate of 0.0001, a batch size of 32 with batch normalization, and 20% dropout. 500 epochs were used for each run with cyclical learning rate and early stopping enabled. Slightly varying these settings (i.e., using a batch size of 16 or a learning rate of 0.005) had little to no effect on performance. Such consistency across small changes in hyperparameters suggests that the chosen configuration was sufficient for training to plateau and efficiently extract the available information from each descriptor.

The results of the model variants are summarized in Table 1. In the following sections, the stand-alone AttentiveFP models will be evaluated to determine the utility of adding the ZINDO-derived electronic descriptors. Next, the AttentiveFP with dense descriptor embeddings is analyzed. Finally, the best performing model is selected for a full analysis. In addition, the Supporting Information contains an investigation into dense-only networks (SI, Section B.3) and test set prediction plots for the different model variants (SI, Section B.4). The relevant code is available from the GitHub repository in ref 56

**Table 1:** Metrics^a^ for All of the Main Variants, with the Best Performing Model in Bold

**Model Variant**	**Validation** *r*	**Test** *r*	**Test MAE (eV)**	**Test RMSE (eV)**
Linear(*E*_ZINDO_)	0.781	0.769	0.304	0.387
Dense(*E*_ZINDO_, MO-RDF)	0.900 ± 0.010	0.920 ± 0.004	0.169 ± 0.006	0.238 ± 0.005
Dense(*E*_ZINDO_, MO-RDF, MFP)	0.897 ± 0.009	0.861 ± 0.003	0.243 ± 0.006	0.315 ± 0.007
MPNN(DA)	0.921 ± 0.008	0.930 ± 0.002	0.157 ± 0.003	0.224 ± 0.004
MPNN(DA, EA)	0.939 ± 0.008	0.946 ± 0.003	0.137 ± 0.005	0.199 ± 0.008
Dense(MPNN(DA), *E*_ZINDO_)	0.939 ± 0.007	0.947 ± 0.004	0.141 ± 0.007	0.197 ± 0.007
Dense(MPNN(DA, EA), *E*_ZINDO_)	0.956 ± 0.005	0.956 ± 0.002	0.121 ± 0.005	0.179 ± 0.05
**Dense(MPNN(DA, EA),** *E*_**ZINDO**_, **MO-RDF)**	**0.956** **±** **0.004**	**0.957** **±** **0.004**	**0.118** **±** **0.004**	**0.178** **±** **0.008**
Dense(MPNN(DA, EA), *E*_ZINDO_, MFP)	0.926 ± 0.006	0.902 ± 0.002	0.201 ± 0.003	0.264 ± 0.003
Dense(MPNN(DA, EA), *E*_ZINDO_, MO-RDF, MFP)	0.930 ± 0.005	0.911 ± 0.002	0.192 ± 0.004	0.253 ± 0.005

a Unique-core validation *r* assesses extrapolation, while core-analogue test set metrics assess interpolation.

### 5.1 Adding Electronic Information to AttentiveFP

To assess the utility of the 4 extra ZINDO-derived electronic terms (μ_*i*_, ϕ_*i*_, $n_{i}^{\mathrm{elec}}$ , $n_{i}^{\mathrm{hole}}$ ), we can compare the performance between the MPNN­(DA) and MPNN­(DA, EA) models. Training the stand-alone AttentiveFP with the default descriptors directly onto *E*_TDDFT_ values, MPNN­(DA), gave a test correlation of *r*_test_ = 0.930, whereas adding electronic atom descriptors in MPNN­(DA, EA) resulted in significantly better performance of *r*_test_ = 0.946. This occurs as the new terms introduce novel electronic information that is directly relevant to the S_1_ energy. We also found that using the stand-alone MPNN with added atomwise electronic terms, MPNN­(DA, EA), gave validation and test correlations of *r*_valid_ = 0.939 and *r*_test_ = 0.946, which was nearly identical to embedding the default AttentiveFP with the molecule-wise ZINDO energy, Dense­(MPNN­(DA), *E*_ZINDO_), further highlighting the link between the ZINDO S_1_ energy and the electronic atom descriptors. The Dense­(MPNN­(DA, EA), *E*_ZINDO_) model used both the electronic atom-level descriptors as well as the molecular-level ZINDO energy embedding, which increased both *r*_valid_ and *r*_test_ to 0.956, showing that an atom-level embedding of electronic information with a global-level embedding of the energy and neural fingerprint introduced the highest proportion of novel information and processed it in the most robust way.

These results indicate the benefit of developing descriptors from excited-state relevant objects, such as transition density matrices, and the importance of correctly incorporating them into versatile neural fingerprints. A further significance of these results is that even if ZINDO data are not available, one can still use the MPNN­(DA) model, which does not require any inputs other than a SMILES string and RDKit connectivity processing to obtain reasonable MAE estimates of 0.157 eV, when compared to the linear fit (0.304 eV) and the current best model (0.118 eV). As such, the pretrained default AttentiveFP model could be used as a rapid initial screening stage before requiring a ZINDO calculation for the slightly more accurate ZINDO-based models.

### 5.2 Accuracy of MPNN-Based Networks with Descriptor Variations

All MPNN-based models drastically outperformed the dense-only variants. The best performing dense variant, Dense­(*E*_ZINDO_, MO-RDF), was significantly better relative to the rest of the dense-only models, obtaining correlations of *r*_valid_ = 0.900 and *r*_test_ = 0.920, showing good performance in interpolative domains. Interestingly, it performed better than Dense­(*E*_ZINDO_, MO-RDF, and MFP), which indicates that the addition of the MorganFP descriptor was detrimental to performance.

Taking the AttentiveFP model with electronic terms and combining it with other descriptors via dense network embeddings offered an optimal configuration to predict $E_{\mathrm{TDDFT}}^{S_{1}}$ target values. As shown in the last 3 rows of Table 1, the MPNN­(DA, EA) model was combined with different embeddings of *E*_ZINDO_, the Morgan fingerprint and the MO-RDF. It shows that both Dense­(MPNN­(DA, EA), *E*_ZINDO_) and Dense­(MPNN­(DA, EA), *E*_ZINDO_, MO-RDF) perform the best with similar *r*_test_ values of 0.956 and 0.957, respectively. Since adding the MO-RDF did not significantly increase performance upon the Dense­(MPNN­(DA, EA), *E*_ZINDO_) model, this suggests that there may be some descriptor redundancy as the novel electronic information introduced by the MO-RDF and ZINDO-derived terms may be very similar. This can be seen in the similar test set prediction plots of these two variants, as shown in the SI, Section B.4.

Adding the Morgan fingerprint caused considerably worse performance. It is conceivable that the connectivity and structural information provided by the Morgan fingerprint are already accounted for by the more advanced neural fingerprint. In this case, including the Morgan fingerprint may dilute the efficiency of training and take away neuron weights that would otherwise be used for more useful information embedding. Furthermore, the binary-bit nature of the MorganFP may not be ideal for concatenation with scalar-type descriptors, as mixing input types can impact model training.^112–115^ One way to overcome this may be to embed the MorganFP and MO-RDF into more compatible latent spaces before combination with other descriptors. This can be achieved using an initial convolutional block or bespoke embedding algorithms^68,116^ where the MO-RDF can further benefit from stacking different MO weightings in different convolutional channels. In a brief attempt to address this, variants were tested where the MO-RDF and MorganFP were processed in parallel using separate dense blocks in order to maintain distinct latent representations before merging them into a final dense block, but this did not result in any meaningful improvement.

### 5.3 Discussion of the Best Performing Model

The final model choice is the Dense­(MPNN­(DA, EA), *E*_ZINDO_, MO-RDF) variant. Applying the 10-fold models to the test set and taking the average of their predicted $E_{\Delta \mathrm{ML}}^{S_{1}}$ values give the overall model prediction, as shown in Figure 5. Calculating metrics for the averaged data gave an $r_{\mathrm{test}}^{\prime }$ of 0.964, an RMSE of 0.161 eV, and an MAE of 0.106 eV, which was a substantial 3-fold improvement over the linear fitting whose MAE was 0.303 eV.

**Figure 5:**
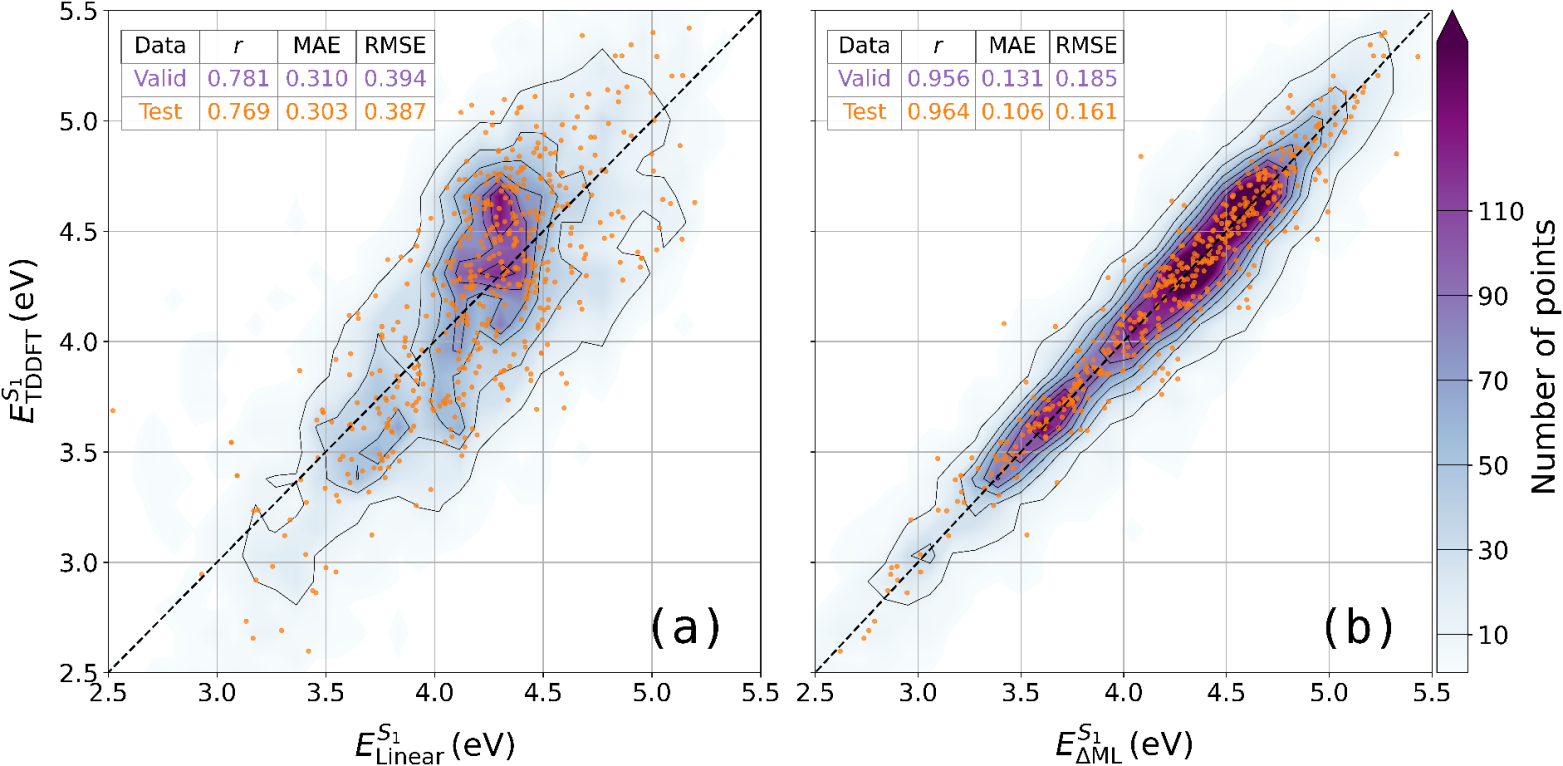
Point density plots showing the distribution of TDDFT values against (a) the ZINDO linear model predictions and (b) the ΔML-ZINDO corrections from the best performing model: Dense­(MPNN­(DA, EA), *E*_ZINDO_, and MO-RDF). Extrapolative validation data is shown as a 2D histogram density heatmap with a 50 × 50 bin grid. The orange points are a random subset of 500 molecules to show the distribution of the test data, without obscuring the validation density. Note: the data in (a) are from the linear model, not the raw ZINDO data which has a 0.5 eV offset as shown in Figure 1. The contour lines correspond to the color bar ticks.

As per Table S3 in Section C.1 of the SI, correlations of $E_{\mathrm{Experiment}}^{S_{1}}$ against $E_{\mathrm{TDDFT}}^{S_{1}}$ for a variety of TDDFT methods range from 0.936 to 0.954, which is comparable to the difference between TDDFT and the ΔML model; thus any further model improvements are not likely to have an impact on virtual screening efforts. The pretrained ML model takes 2 ms to perform a prediction per molecule, a ZINDO calculation takes approximately 2 s, while a TDDFT calculation takes approximately 20 min, i.e., ML only adds an extra 0.1% cost to ZINDO. As such, we have successfully mapped S_1_ ZINDO energies onto the more accurate and expensive TDDFT energies at the cost of a semiempirical ZINDO calculation.

By plotting the 9500 test set prediction errors between the ΔML and linear models, we can see how they perform across the full range of interpolative TDDFT values, as shown in Figure 6. In (a), we see the distribution of prediction errors, where 95% of ML predictions had less than 0.32 eV of error and 95% of linear predictions had less than 0.73 eV of error. The maximum |Δ*E*_ML_| value was 2.0 eV against the maximum |Δ*E*_Linear_| which was 2.7 eV, while the average median |Δ*E*_ML_| was 0.07 eV and median |Δ*E*_Linear_| was 0.26 eV. In (b), it shows binned intervals of the true TDDFT energies and the median errors between the ML and linear model associated with each interval. We see that the ML model performs consistently across the whole distribution with median errors ranging from 0.05 to 0.22 eV at most. The linear model is much less consistent, with median errors greater than 0.60 eV in the outermost regions.

**Figure 6:**
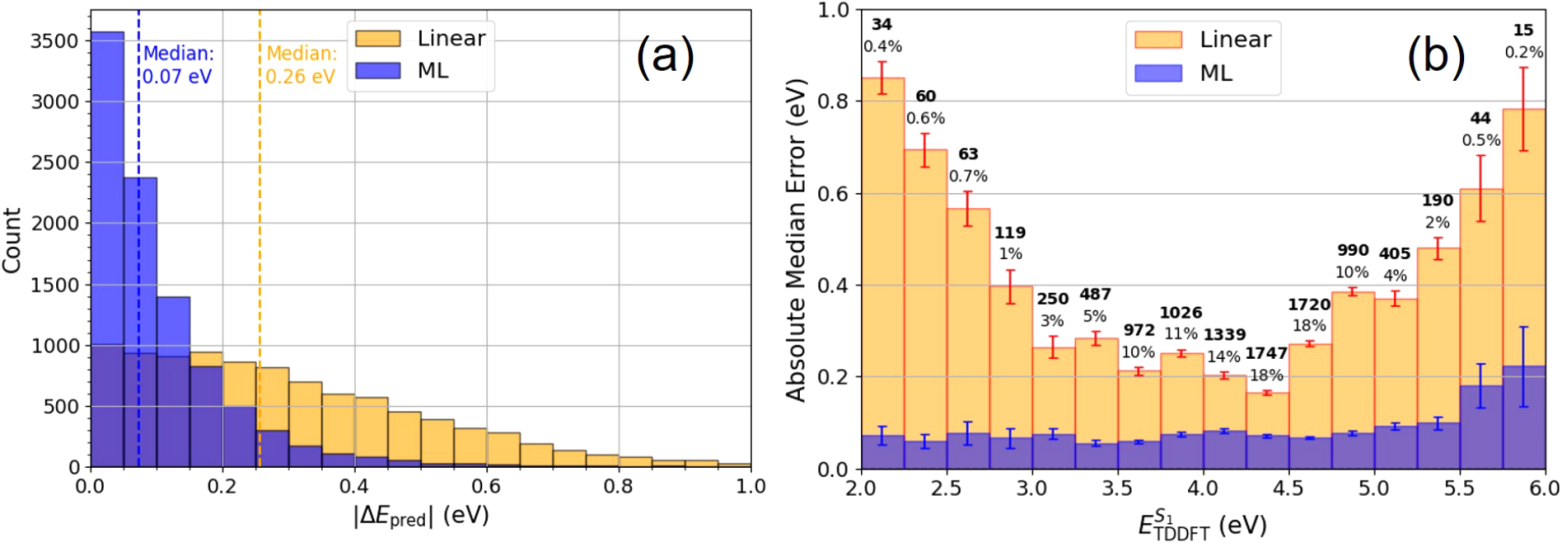
Analysis of ΔML and linear fit prediction errors: Δ*E*_pred_ = *E*_pred_ – *E*_TDDFT_. (a) The 9500 test data distribution of absolute errors between predicted and true TDDFT values, where 95% of ML predictions have an error of less than 0.32 eV. (b) Median error for different intervals of TDDFT energy. The linear model performs worse for edge cases, whereas the ML model is consistent across the entire range. Molecule counts are shown above each bar.

The ML model has great reliability as it is significantly less sensitive to outlier molecules and offers remarkable improvements within the visible range $\left(1.7<E^{S_{1}}<3.3\;\mathrm{eV}\right)$ . This is particularly convenient if one is searching for low-energy emitters, which are sought after in a range of applications, such as organic semiconductor devices.^117–121^ For example, if one is interested in identifying molecules with up to 25 heavy atoms absorbing in the visible region, one expects to find ≃6% of molecules with such characteristics (based on our testing data set). The linear ZINDO model only identifies 45% of those molecules as being within that range, of which only 38% are predicted with less than 10% relative error, while ΔML-ZINDO identifies 86% correctly with 91% having less than 10% error.

As a final evaluation of the model, the 9500 test set was sorted in ascending order by heavy atom count (HAC) and every 19th molecule was selected to form a 500 molecule HAC sample. For this HAC sample, using the same BLYP35/3-21G* optimized geometries, TDDFT single-point calculations were performed at the M06-2X/def2-TZVP levels so their S_1_ energies could be compared to the ΔML-ZINDO model predictions, as shown in Figure 7. Panel (a) shows excellent correlation between our original target level of theory (3-21G*) and the considerably more accurate (def2-TZVP) basis set, which in panel (b), we can see the ΔML-ZINDO model has inherited. Moreover, as shown in Sections C.1 and C.2 of the SI, 3-21G* and def2-TZVP have a good correlation to experimental values by approximately 0.95. The results for other method pairs on the HAC sample are available in Section C.3 of the SI.

**Figure 7:**
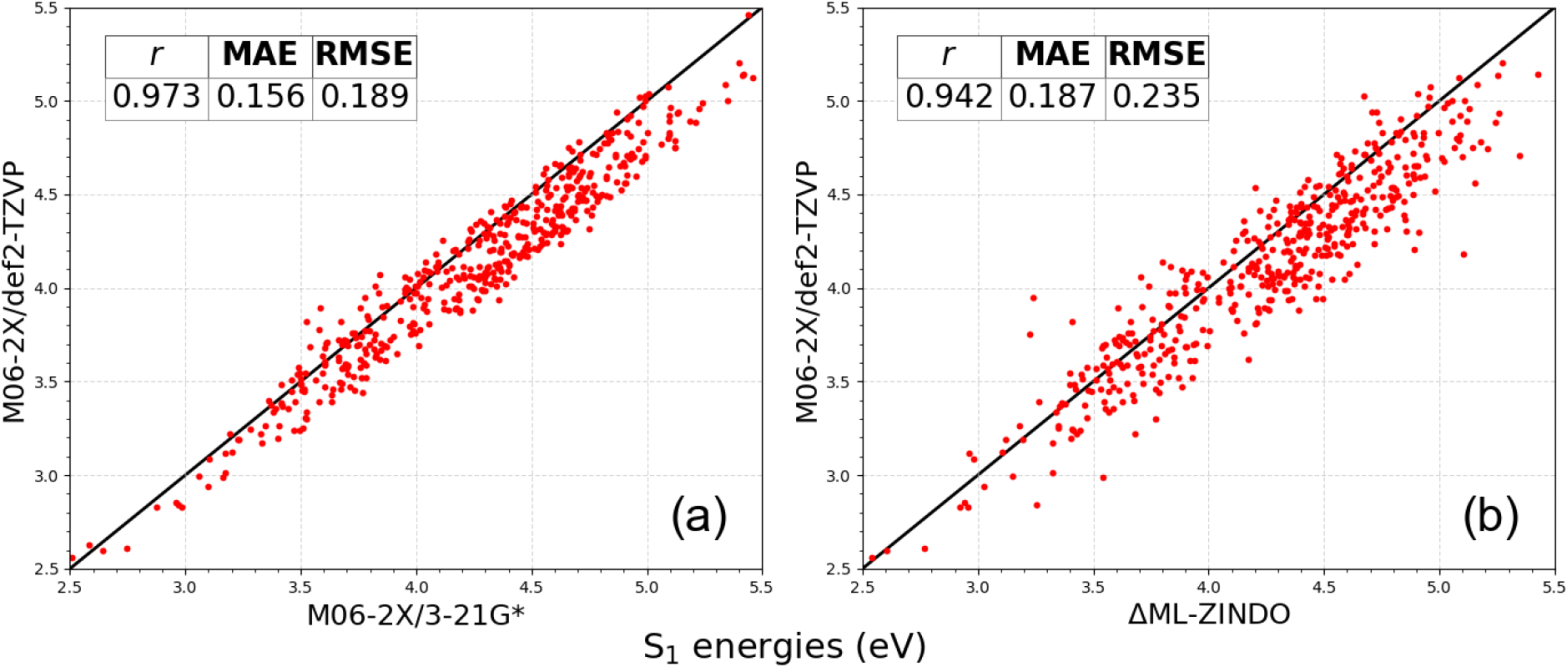
S_1_ energies for a 500-HAC sample comparing M06-2X/3-21G* (a) and ΔML-ZINDO (b) to the more accurate def2-TZVP basis set.

To summarize, we have shown the following: (1) The correlation between ΔML-ZINDO and M06-2X/3-21G* is *r* = 0.95, as applied to our 9500 test set. (2) The correlation between M06-2X/3-21G* and M06-2X/def2-TZVP is *r* = 0.973, as applied to the HAC sample. (3) From two previous works,^44,51^ the correlation between M06-2X/def2-TZVP and experiment was shown to be *r* = 0.95 (excluding solvent effects). Consequently, although direct experimental values are unavailable for the specific 500-HAC sample, the established correlations indicate that ΔML-ZINDO approximates S_1_ energies with sufficient accuracy to guide screening campaigns. Ultimately, the optimal strategy depends on the balance between data set size, computational resources, and error tolerance. While standard DFT workflows remain viable for smaller libraries (up to ∼10,000 molecules), the ΔML-ZINDO tool offers a distinct advantage for high-volume data sets (from 10^5^ to >10^6^ molecules) where other methods become intractable. Thus, the model is best utilized as a high-throughput prescreening step to efficiently filter candidates before applying more computationally expensive levels of theory.

### 5.4 Application to External QCDGE Data Set

To illustrate the generalizability of the framework to external data sets where conjugated-core clustering has not been preapplied and different combinations of low- and high-level calculation pairs are considered, we trained a new Dense­(MPNN­(DA, EA), *E*_ZINDO_, MO-RDF) model on the QCDGE data set.^55^ This data set records first singlet excited-states data at the ωB97X-D TDDFT level, with the larger 6-31G* basis set. The total data set consists of 443k molecules, from which we utilized the PubChemQC subset of 92k molecules containing exactly 10 heavy atoms. This 92k set was filtered to only retain molecules with 1 or 2 rings, while nonaromatic carbocycles (alicyclic), including fused alicyclic polycycles, were removed. This was done to reduce the total data generation time while ensuring relevance to organic electronics by biasing the set toward more electronically delocalized and conjugated structures, leaving 48k molecules. These remaining molecules were then ordered by their database IDs and every odd-numbered molecule was put into the training set and every even-numbered molecule was put into the testing set, such that each set had ∼24,000 molecules.

To generate our model input data and ensure consistency in structural conformations, we applied a PM7 geometry optimization to the B3LYP/6-31G*-BJD3 preoptimized geometries from the QCDGE data set, then performed ZINDO and extracted the particle-hole densities. As per the main analysis, we applied 10-fold cross-validation and retrained our models on ZINDO data to predict the ωB97X-D/6-31G* TDDFT target. We applied the resultant optimized network ensemble on the QCDGE-derived test set, where the results are shown in Figure 8. We see in panel (a) that the TDDFT S_1_ energies against ZINDO predictions had a correlation of 0.880, while in panel (b), ΔML-ZINDO predictions achieved a correlation of 0.993, which was an improvement of 13% over ZINDO, with a 10-fold reduction in mean absolute error. Results and the distribution of data were nearly identical for the QCDGE validation set.

**Figure 8:**
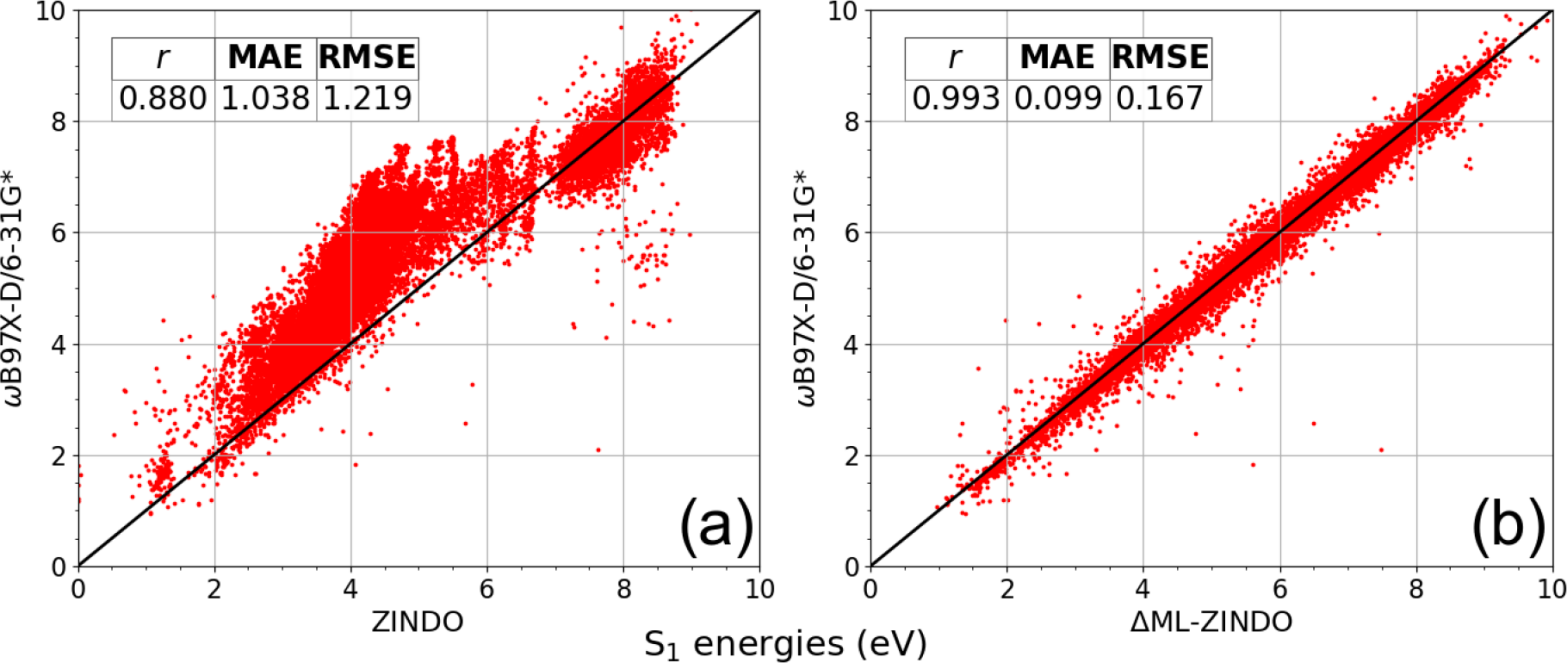
Results from retraining Dense­(MPNN­(DA, EA), *E*_ZINDO_, MO-RDF) on the 48k filtered subset of the QCDGE data set. This shows predictions on the 24k test set, for (a) ZINDO and (b) ΔML-ZINDO against the ωB97X-D/6-31G* TDDFT target.

The model attained a significantly higher correlation on the QCDGE subset (0.993) compared to that of our ZINC-derived test set (0.954). We attribute this to the significantly lower electronic diversity of the QCDGE subset, which was caused by the restrictive chemical space of the 10-HAC limit. Capping molecules at exactly 10 heavy atoms severely reduces the possible variations of unique conjugated cores in the data set. We applied our common-core clustering algorithm to the QCDGE subset and found it was heavily skewed toward small fragments and “singleton” clusters. Of the 48k molecules, 35k had small common conjugated units with fewer than 6 heavy atoms and were subsequently discarded by the clustering algorithm due to limited conjugation. The remaining 13,000 molecules formed 2175 clusters, 1414 of which were singleton clusters (containing only one molecule) leaving only ∼700 unique core clusters with one or more analogues per cluster. This skewed unique core distribution would not be sufficiently resolved even if we trained on the full 443k molecules. This means the QCDGE data set has a significantly reduced electronic diversity compared to the 7602 unique cores (with up to 25 heavy atoms) that form our in-house training set. Consequently, the higher performance on the QCDGE data set is a result of the model interpolating within a significantly narrower electronic space.

### 5.5 Adaptation for Oscillator Strength Classification

While not the focus of this work, we also adapted our model for S_1_ oscillator strength prediction, where we achieved an improvement over ZINDO by 35% (correlation 0.579 to 0.779) for the validation set and by 60% (0.524 to 0.839) for the test set. To quantify the model’s utility for virtual screening, we evaluated its classification performance^122^ using an oscillator strength threshold of *f* > 0.4, as shown in Figure 9. The method achieves a recall of 70.3%, meaning it successfully identifies the majority of highly emissive molecules. Crucially, the model demonstrates a high precision of 82.3%, implying that when a molecule is predicted to be bright, it is highly likely to be a true positive with a specificity of 96.5%. Overall, the method correctly classifies the emissivity of the molecules with an accuracy of 91.5%. Further details on the adaptation for oscillator strength prediction are reported in Section D of the SI.

**Figure 9:**
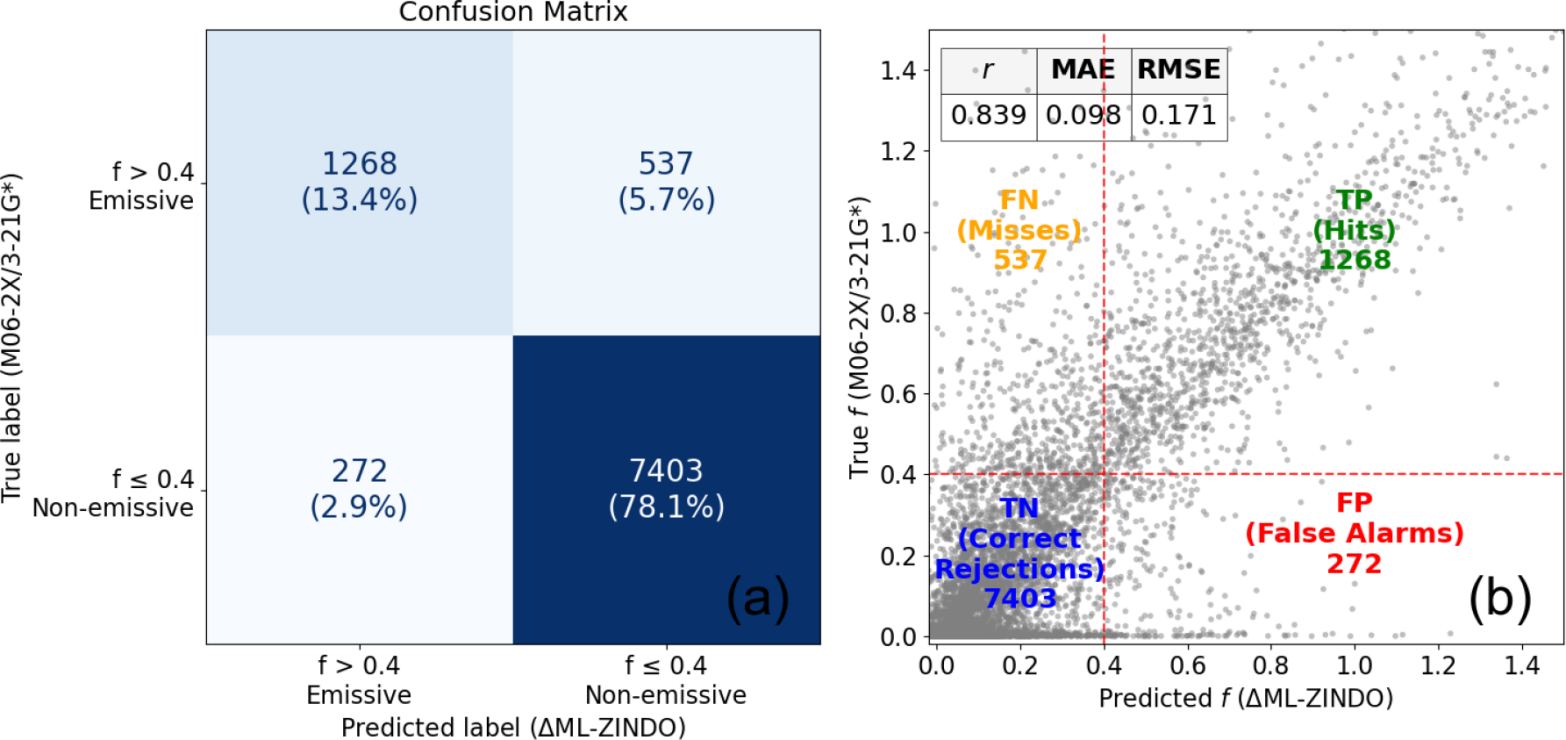
Classification and regression performance of ΔML-ZINDO for S_1_ oscillator strengths (*f*) on the test set. (a) Confusion matrix using a threshold of *f* > 0.4 to classify molecules as potentially emissive. (b) ΔML-ZINDO predictions against the TDDFT oscillator strengths. The plot is divided into quadrants corresponding to the confusion matrix: true positives (TP), false negatives (FN), false positives (FP), and true negatives (TN).

## 6 Conclusion

In conclusion, we have shown that a representative data set of excited-states calculations at the ZINDO level can be improved to approach the accuracy of more expensive TDDFT calculations by developing machine learning models that correct for molecule-specific systematic errors of the semi-empirical method. The proposed method trained MPNN neural fingerprints with ZINDO-derived electronic atom descriptors and further dense network embeddings on a carefully curated data set of 7600 π-conjugated systems. This was used to predict the S_1_ energies of a 9500 molecule test set to a correlation of 0.964, which is consistent with the correlation between different levels of TDDFT. We demonstrated the generalizability of the model by adapting it for a different high-level method, achieving a correlation of 0.993 on a subset of the QCDGE data set. Furthermore, we modified the architecture for oscillator strength prediction, where it successfully improved ZINDO predictions from 0.524 to 0.839. As such, we have shown that our ΔML-ZINDO model approximates S_1_ energies and oscillator strengths with sufficient accuracy to guide screening efforts and efficiently filter candidates before applying more computationally expensive levels of theory.

These results convey the significance of introducing electronic information relevant to the prediction of excited-state energies and highlight how one can make further use of the output of low-level QC calculations to obtain multiple crude estimators of the target property within a ΔML framework. Although TDDFT is generally thought of as only moderately expensive, the cost still becomes significant in high-throughput studies that aim to screen chemical data sets with millions of molecules. This electronically informed ΔML approach is an effective, lightweight tool that makes it viable to computationally screen extremely large databases and search for properties across the whole distribution due to its robustness against outliers. With the pretrained model requiring only 2 ms per molecule compared to the 20 min typically required for TDDFT, the ML correction is sufficient to transform ZINDO into a very useful virtual screening tool at a negligible additional cost. Consequently, ΔML-enhanced ZINDO makes such goals tractable and will be an important tool to integrate into big-data infrastructure.

## Supplementary Material



## Data Availability

We provide the data set, pretrained models, and PyTorch-based training tutorials to reproduce the work in a public GitHub repository.^56^

## Notes

The Supporting Information contains 4 sections (A, B, C, D): Section A covers the algorithm used for conjugated core clustering, the training set processing steps, test set formation, and a similarity plot analysis; Section B covers further evaluation of descriptors, a full analysis of Dense-only networks, and some additional test set plots; Section C contains a rigorous cross-validation to evaluate our choice of TDDFT/3-21G* method against TDDFT/def2-TZVP and experimental values; Section (D) shows the details of adapting our model for oscillator strength prediction (PDF)
