# Supplementary material for: Predicting S1 TDDFT Energies from ZINDO Calculations Using Message-Passing ΔML with Electronically Informed Descriptors
Source: J Chem Theory Comput. 2026 Jan 22;22(3):1476–90. doi: 10.1021/acs.jctc.5c01587 (PMC12895415; doi:10.1021/acs.jctc.5c01587)
Supplement: Supplementary file 1 [file ct5c01587_si_001.pdf]

Predicting S_1 TDDFT energies from ZINDO calculations using Message-Passing ΔML with electronically-informed descriptors

Adam Coxson, Ömer H. Omar, Marcos del Cueto, and Alessandro Troisi

Department of Chemistry, University of Liverpool, L69 7ZX, UK

Supplementary Information (SI)

A ZINC-derived dataset clustering, filtering and train-test splits

A.1 Common-core clustering algorithm

The clustering algorithm used to define the dataset of this manuscript was originally used in ref. [1] to accelerate the evaluation of excited states for a set of 12M molecules within the (2015) ZINC database [2] with TDDFT. It was based on the assumption that molecules with similar conjugated core (defined rigorously below) have similar electronic properties. Thus it is only necessary to calculate one representative molecule for each group of molecules with the same conjugated core to gain an understanding of the electronic structure across the entire group.

The clustering algorithm is as follows:

- Extract SMILES strings and ZINC ID for all 12 million molecules from database.
- Retain all atoms with formal charge or that are sp^2-hybridised on the basis of their connectivity (i.e. a carbon bonded with three atoms)
- The heteroatoms oxygen, nitrogen and sulphur are retained whenever they are connected to two sp^2-hybridised atoms.
- Halogens, nitrile, hydroxyl, phenyl and aldehyde groups are removed.
- Only the largest conjugated fragment is retained if the molecules consist of more than one conjugated fragment. This is defined as the conjugated core.
- Common core smiles with less than 6 heavy atoms are discarded. The smallest common core conjugated unit accepted is a benzene ring.
- The smallest molecule of each set of cores is selected according to the shortest SMILES, as shown in Fig. S1. This is the 'representative' molecule of the cluster.

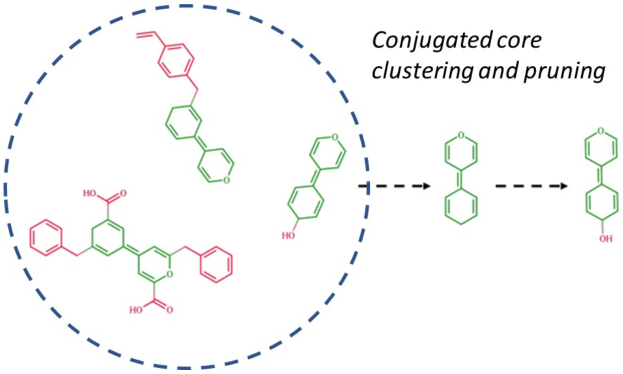

Figure S1: Diagram of the clustering that was performed to identify a unique conjugated cores across many molecules (green). For each cluster of cores, the one with the shortest SMILES [3] is chosen as the representative core of that set.

There are only 150,000 distinct conjugated cores from the initial dataset of 12 million molecules, suggesting that sampling the space of unique cores can be an efficient way to explore the chemical space for excited state properties by avoiding the inclusion of many similar molecules. To verify the validity of this hypotheses we considered a set of 650 pairs of different molecules sharing the same conjugated core (the smallest molecule and another random molecule) and evaluated the vertical excitation energy of each molecule at the M06-2X/3-21G* TDDFT level. As shown in Fig. S2a, the deviations between S_1 energies (ΔE_{S_1}) for 69% of pairs is less than 0.1 eV, and for 87% is less than 0.3 eV, which highlights the similarity between the S_1 energies (and hence electronic structure) of core-analogues. A similar distribution is found for ΔE_{S_2} in (b).

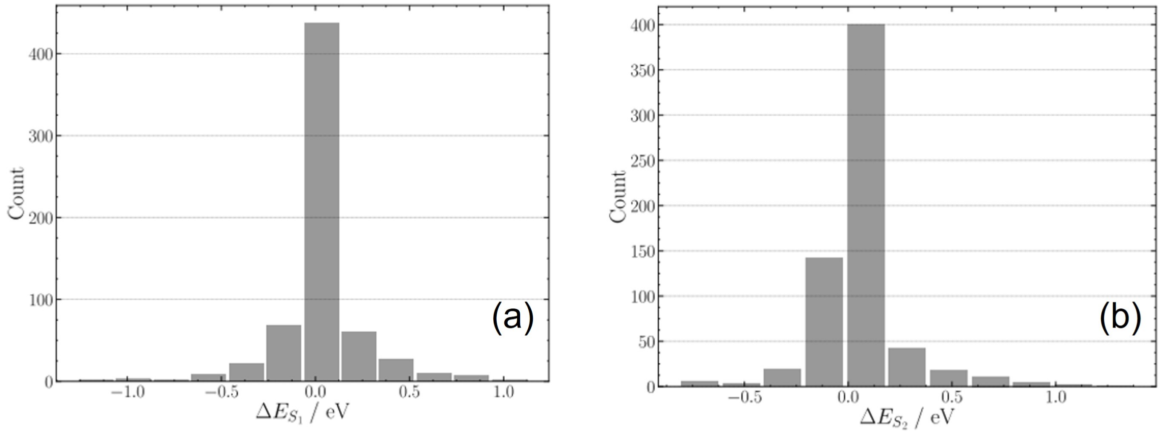

Figure S2: First (a) and second (b) singlet state excitation energy deviations (ΔE_{S_1}, ΔE_{S_2}) between 650 core-analogue pairs. In (a), only 69% of pairs deviate by 0.1 eV, which indicates the similarity of low-lying electronic structure between core-analogues.

A.2 Training set processing and validation

The common-core clustering formed the ZINC-derived unique-core dataset of 150,000 molecules. This was significant as it optimised the dataset for electronic structural diversity, which is not necessarily guaranteed even if chemical structural diversity is high. During the initial stages of model

development and prototyping, only a small subset of the full dataset is needed, so to further restrict the training data to a more appropriate size, the 150k were reduced to 9050 molecules by selecting for molecules with 10 to 25 C, N, O, S, F heavy atoms. Next, compounds containing the nitroso (-N=O) group were removed as they consistently had S_1 energies centred around 3.3 eV, which made them easy to predict during training and arbitrarily inflated overall model performance by up to 10%. It was necessary to reduce this dataset and remove these molecules so model development can be more efficient and focus on a more representative set of molecules. Nevertheless, the size of the dataset can be expanded by adding core-analogues or changing constraints on the number and type of heavy atom, so that future iterations of the model can be trained on larger subsets. Note, we have not identified or removed any outlier molecules from our training set. The final training data set comprised of 7602 molecules containing only H, C, N, O, S, and F atoms, with unique conjugated cores and 10-25 heavy atoms.

During model training we apply k-fold Cross Validation (CV) [4] using 10 training folds with 10 different initialisations of neural networks within a network ensemble. Note, standard k-fold CV uses k models with the *same* network parameter initialisations, whereas we ensure each fold model has a different initialisation seed, thus maximising stochasticity via randomisation of both validation folds and initial parameters. This 'k-fold ensemble with random initialisations' is only necessary as we assume the distributions across all training and validation folds are sufficiently homogenous. This means that each fold model is trained on 6840 unique cores, and validated on 760 unique cores not seen during training (hence unseen electronic structure). After training, all 10 validation fold predictions are combined to obtain predictions for the full training set, which we denote as the validation set. This means we have a good idea of model performance across the validation set, which shows how well we can predict novel, unseen conjugated-cores.

A.3 Test set split

We define the term *core-analogues* to describe two different molecules with the same conjugated core but different overall chemical structures, differing by terminal functional group, but not extensions of their conjugated parts. Comparing the unique cores in the training set, we can identify the number of analogues each training-core has in the original set of 12 million molecules. Of the training cores, approximately 30% are completely unique, with no core analogues at all. 20% of the training molecules had one analogue, 17% had 2, and 33% had three or more analogues. This means that 70% of molecules in the training set had at least one molecule with a matching core from the original set of 12M. We formed the test set of 9500 extended π-conjugated molecules of up to 35 heavy atoms by sampling up to a maximum of 3 analogues per training set molecule. This meant that for each molecule in the test set, there was exactly one molecule in the training set that had the same conjugated core. An illustration is shown in Fig. S3, where molecule A represents a core with no analogues, B represents one of the 20% of molecules with exactly one core-analogue, C has 2 analogues in the test set, while D has 3 in the test set. When obtaining statistics for the testing set, we apply the full 9500 molecule test set to each pre-trained fold-model (there are 10 fold models in the ensemble) and then average these 10 duplicate prediction sets to obtain an overall ensemble prediction, which gives us a mean and standard deviation as a measure of model uncertainty.

The organisation of our dataset into these validation and test sets based on conjugated core is atypical for most machine learning, where no other datasets are organised in this way. However, this is a particular strength and feature of this work. The validation data contains unseen π-conjugated

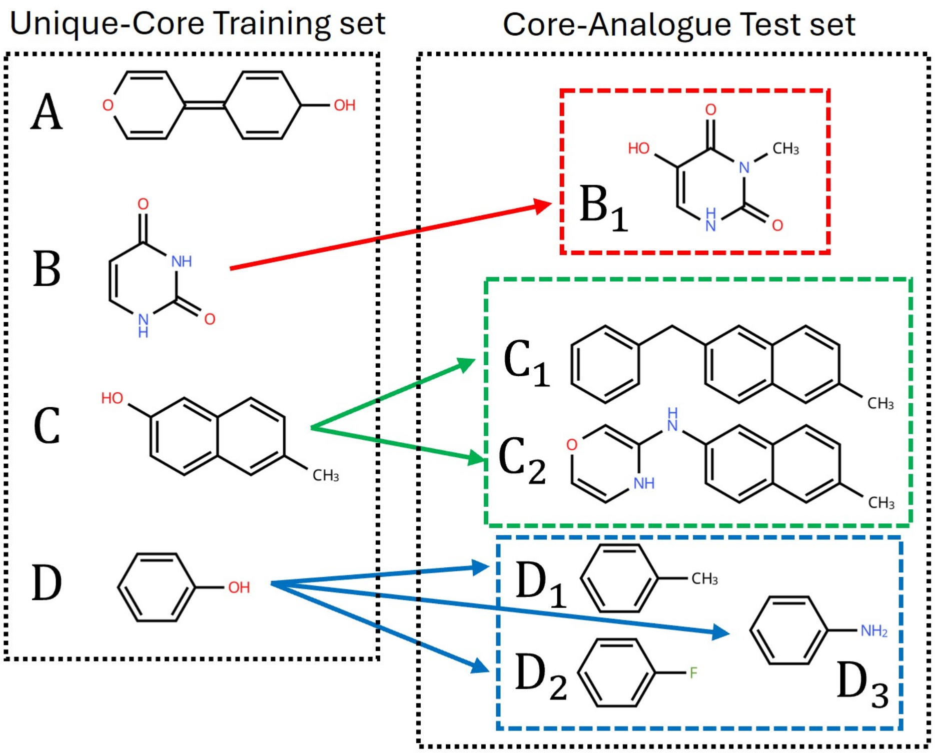

Figure S3: An illustration of common-core one-to-many mapping between the train and test set. The training set contains distinct, unique conjugated core molecules, while the testing set contains up to 3 matching core-analogues for a given training-core (but not the training core itself). In this example, molecule A has no analogues in the test set, B has 1, C has 2 and D has 3. Note, this diagram is demonstrative and does not necessarily reflect the molecules in our dataset.

cores, each with a unique electronic structure, which effectively tests the models generalisability towards out-of-domain data (extrapolation). While molecules with the same conjugated core likely share a similar electronic structure, this does not necessarily mean they share a similar geometry; core-analogues differ by terminating groups (possibly doubling in size). As such, the test set evaluates in-domain data (interpolation), as the model has seen the same conjugated core during training, but the overall structure of the corresponding test set molecule may be significantly different. In the context of machine learning, this means the test set effectively evaluates the models ability to interpolate to similar electronic structures while extrapolating to and accounting for significant variations in geometry.

In other ML works, the validation set is usually interpolative while the test set is more extrapolative, but this is often done according to some application, element or functional group specific factor, rather than the diversity of the electronic structure, which we have optimised for in this work via the clustering. For this reason, both validation set and testing set metrics are reported to give an indication of model performance under both extrapolative and interpolative conditions. As shown in Section 5 in the main manuscript, we obtain similar performance (for our best model) for our validation and test sets, which indicates excellent generalisability of our framework.

A.4 Similarity plot analysis

To give a standard similarity plot analysis, we have calculated a variety of fingerprint/distance pairs for our training and testing set: The Morgan/Tanimoto [5], Atom Pair/Cosine [6, 7] and RDKit/-Cosine [8] similarities. The Morgan/Tanimoto distance is included as it is a very common similarity metric, RDKit and Atom Pair similarities were included as an analysis by Duke et. al. [9] found that both of these pairs performed well at classifying electronic properties. Moreover, to show the utility of the MO-RDF as a fingerprint, we also provide the MO-RDF/Cosine similarity metric.

Metrics of the similarities are provided in Table S1. These pairwise similarities were plotted against difference in S_1 energy across all pairs of molecules for our 7602 molecule training set, as shown in Fig. S4 and 9500 molecule testing set, as shown in Fig. S5. An ideal electronically-informed similarity metric would define a diagonal distribution from high ΔE and low similarity to low ΔE and high similarity. From the analysis, we make two main observations: (1) The distribution of energies and similarities are near identical for the train and test split, indicating the homogeneity of structural environments between these splits. (2) The distribution of Atom-Pair/Cosine and RDKit/Cosine similarities are centred around a similarity of 0.4, whereas the distribution of MO-RDF/Cosine similarities is centred around 0.7 and skewed more diagonally to the lower right, signalling higher similarity for molecules with smaller differences in S_1 energies. This potentially indicates that the MO-RDF has greater sensitivity to differences in electronic structure than other metrics (that are more strictly based on chemical connectivity). However, upon visual inspection this effect appears small, and possibly suggests a limitation in representational power of the MO-RDF (despite being slightly better at this than the other metrics).

Table S1: Similarity distribution statistics for training and testing sets. The 25% and 75% columns represent similarities around the 0.25 and 0.75 quartiles. The training and testing sets have 28,891,401 and 44,930,460 molecule pairs, respectively.

**Metric**	**Mean**	**Median**	**Std Dev**	**Min**	**25%**	**75%**	**Max**
Unique-core training set							
MorganFP/Tanimoto	0.126	0.118	0.049	0.000	0.092	0.151	0.917
Atom-Pair/Cosine	0.429	0.432	0.104	0.000	0.360	0.500	1.000
RDKitFP/Cosine	0.390	0.393	0.092	0.000	0.329	0.453	1.000
MO-RDF/Cosine	0.700	0.720	0.139	0.003	0.623	0.799	1.000
Analogue-core testing set							
MorganFP/Tanimoto	0.127	0.120	0.049	0.000	0.093	0.153	1.000
Atom-Pair/Cosine	0.422	0.426	0.099	0.000	0.357	0.490	1.000
RDKitFP/Cosine	0.407	0.407	0.095	0.000	0.342	0.472	1.000
MO-RDF/Cosine	0.705	0.724	0.137	0.002	0.627	0.804	1.000

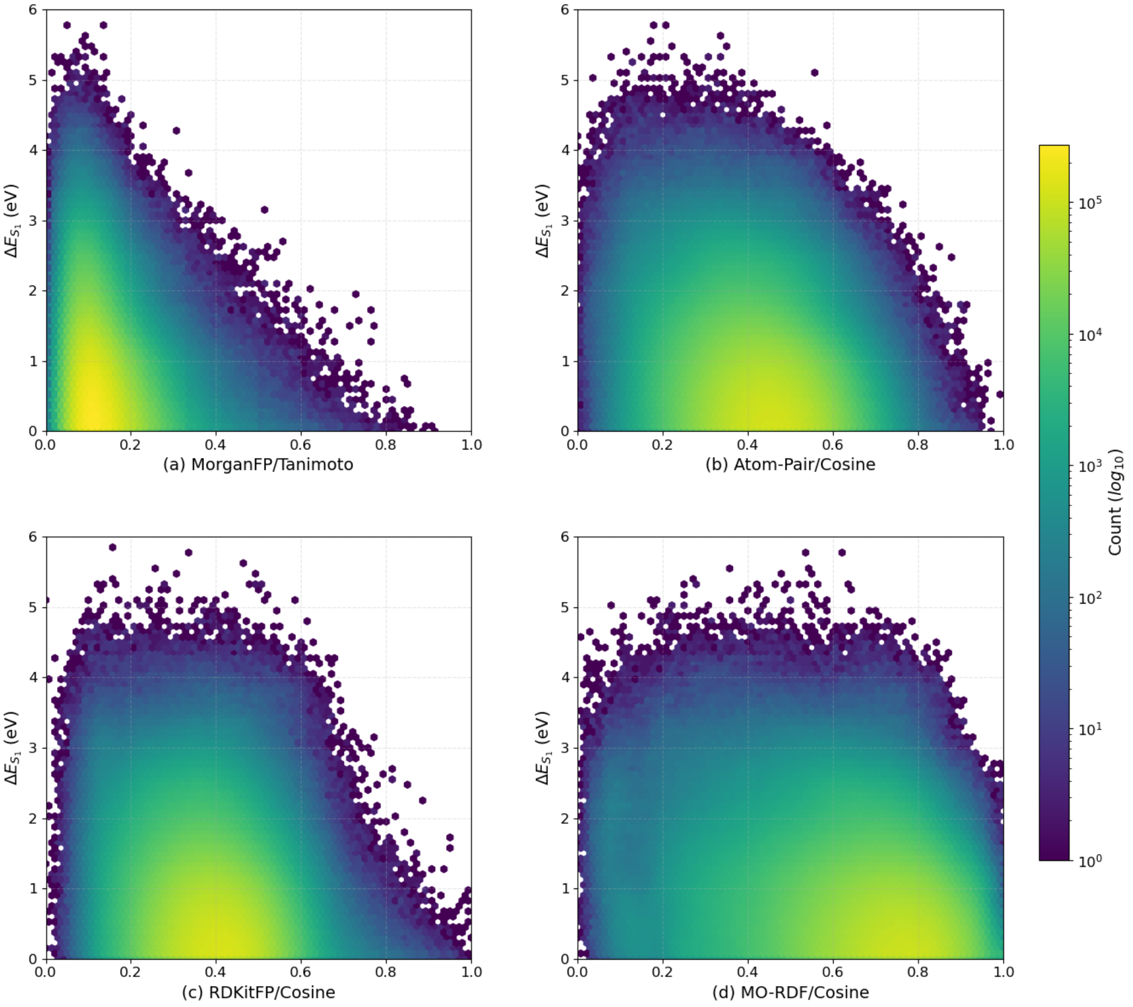

Figure S4: For the training (validation) set, plots of difference in S_1 energy against molecular pair similarity for (a) MorganFP with Tanimoto distance, Atom-Pair (b) and RDKit (c) with cosine distance, as well as the electronically informed MO-RDF, which can also act as a similarity metric when paired with a distance function (cosine). The colour bar indicates the number of pairs per hexagonal pixel.

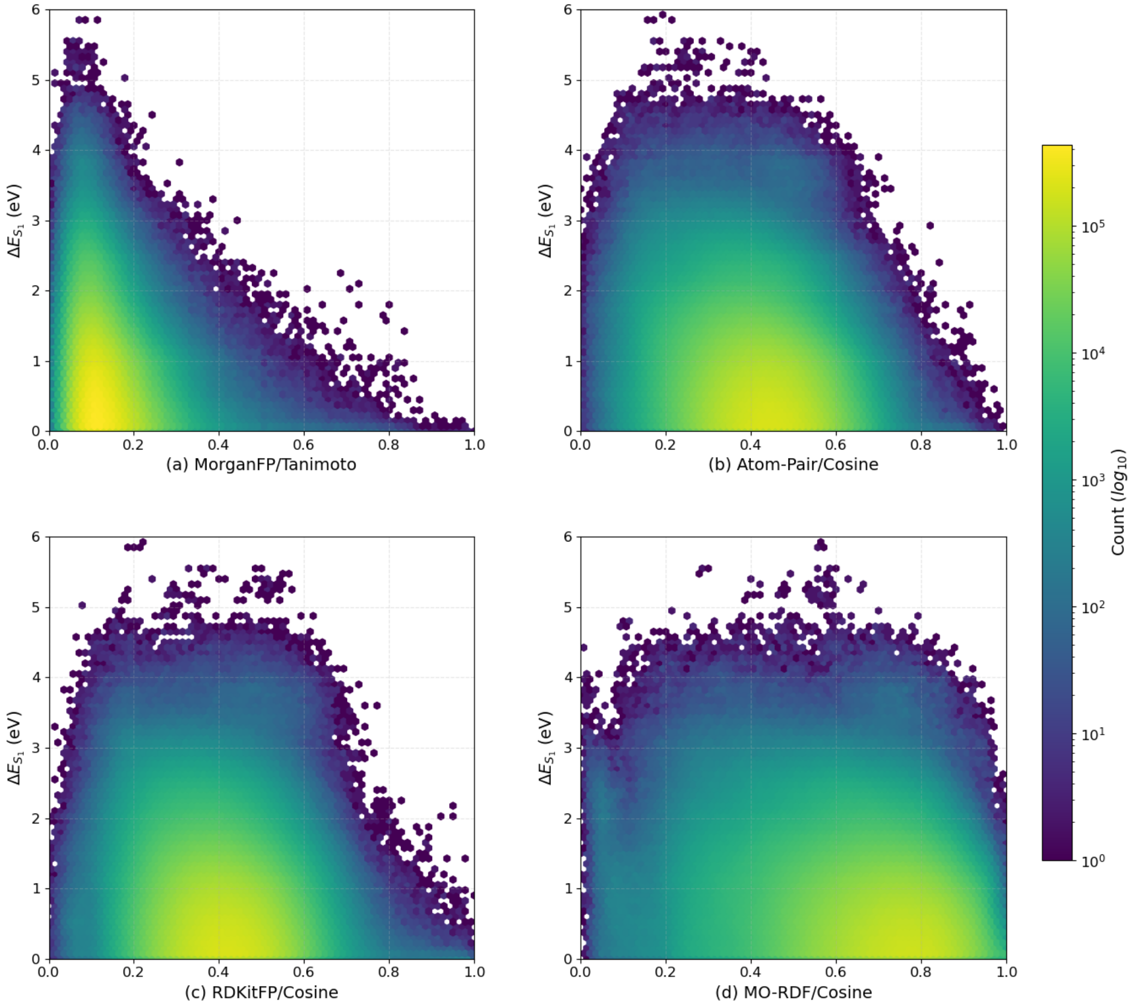

Figure S5: For the testing set, plots of difference in S_1 energy against molecular pair similarity for (a) MorganFP with Tanimoto distance, Atom-Pair (b) and RDKit (c) with cosine distance, as well as the electronically informed MO-RDF, which can also act as a similarity metric when paired with a distance function (cosine). The colour bar indicates the number of pairs per hexagonal pixel.

B Further evaluation of descriptors and architectures

B.1 Mulliken charges and atomic potentials as descriptors

The Mulliken charges and atomic potential based terms are good descriptors of the electronic structure and have been useful features in machine learning endeavours [10–13]. This can be further illustrated by using them to characterise the distribution of on-diagonal elements of the Fock matrix [14], as shown in Fig S6. Simply plotting the Fock elements for the carbon 2s2s atomic orbital self-interaction against the carbon's Mulliken charge and atomic potential allows us to characterise different regions. As can be seen in the insets, the different regions correspond to different functional groups, i.e. all points in the upper-left-most region correspond to the central carbon of methyl groups. Note, the data in this plot were extracted from Fock and density matrices from B3LYP/3-21G* calculations of molecules in the dataset, but were not part of the calculation data used to train ML models in this work.

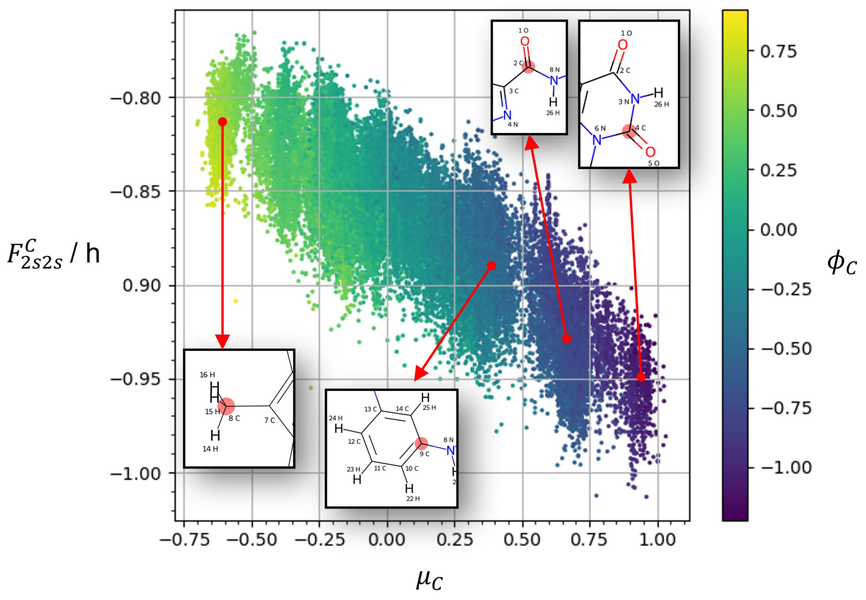

Figure S6: Plot of onsite carbon 2s2s Fock element self-interactions (F^C_{2s2s} in hartrees) against the carbon's Mulliken charge (μ_C), with the atomic potential (ϕ_C) as the colour heatmap. This shows how these two variables are effective descriptors of the electronic structure, as it clearly characterises the carbons according to their functional group, as per the insets.

B.2 Dense networks

Dense networks consist of a series of fully-connected layers of artificial neurons, which result in a highly parameterisable, nested non-linear function. This enables neural networks to act as universal approximators of any given function. The input layer consists of a vector x with length N_0 and the output layer consists of a single node. The output of the i-th neuron in hidden layer l y^l_i can be written as

$$y^l_i = a(\boldsymbol{w}^l_i \boldsymbol{x} + b), \tag{S1}$$

where a represents a non-linear activation function, and $\boldsymbol{x}$ is the input vector from the initial layer or previous hidden layer. The learnable free parameters are the bias b and the weight vector w that operates element-wise on $\boldsymbol{x}$. An entire layer can be represented by

$$f_l(x) = a_l(\mathbf{W}_l \boldsymbol{x}_{l-1} + \boldsymbol{b}_l), \tag{S2}$$

where the matrix $\mathbf{W}_l$ represents all of the weight vectors that fully connect the output from the previous layer x_{l-1} with the neurons in the current layer. This in turn can be recursively applied as a series of nested non-linear functions to represent a 3-layer dense network;

$$y = f_3(\boldsymbol{f}_2(\boldsymbol{f}_1(\boldsymbol{x}))). \tag{S3}$$

B.3 Evaluating descriptors using Dense networks

We can somewhat quantify the proportion of novel information contained within descriptors by evaluating the performance of singular descriptors, E_{ZINDO}, MO-RDF, and MorganFP (MFP), and their groupings. Dense-only networks can only be applied to molecule-level descriptors, providing no mechanism for introduction of the ZINDO-derived atom-level information. Consequently, different descriptions of the first-excited states are necessary, which is why MO-RDFs were designed.

As shown in Table S2, different variations of dense networks were analysed, where class X models operate on a single descriptor and class Y models operate on descriptor set groupings. The E_{ZINDO} equivalent essentially recreates the linear fitting using the networks. Applying the Dense(MFP) model to directly predict E_{TDDFT} resulted in worse r_{valid} and r_{test} than the linear fitting. However, considering the Morgan fingerprint is a highly-sparse binary bit vector of the structure and contains no specific electronic information, it is still impressive that the model is able to achieve $r_{test} > 0.5$. The MO-RDF obtains comparable results, suggesting that it contains enough structural and electronic information for a moderate correlation. Moving onto class Y models, combining the MorganFP with the MO-RDF gave slightly better correlations than their individual variants, which suggest that each descriptor brings in unique information that can be embedded into the latent space. The Dense(E_{ZINDO}, MO-RDF) model performed the best, achieving test correlations of up to 0.920 ± 0.004, which is a significant improvement upon the linear fitting of 0.769.

While dense-only networks are consistently outperformed by the MPNN-based models, they obtain at least moderate positive correlations (0.5 to 0.8), even with only single descriptor variants. Despite the simplicity of dense networks, they are still capable of extracting and embedding a sufficient amount of information, suggesting their utility in descriptor performance evaluation, virtual screening tools, or as part of larger, more advanced frameworks. The dense-only results from this work suggests that the MorganFP and MO-RDF are viable candidates for testing within MPNN-based models.

Table S2: Basic Dense networks with one descriptor (class X) or a combination (class Y) of E_{ZINDO}, the Morgan fingerprint (MFP), and MO-RDF. Models are applied to the 9500 analogue test set, where correlations are predicted for each fold model and then averaged together. Only models that utilise E_{ZINDO} terms had residual connections. All hyperparameters matched the standard set.

**Model Variant**	**Validation r**	**Test r**	**Test MAE (eV)**	**Test RMSE (eV)**
Linear(E_{ZINDO})	0.828	0.769	0.306	0.388
Dense(E_{ZINDO})	0.786 ± 0.020	0.774 ± 0.001	0.300 ± 0.002	0.393 ± 0.003
Dense(MFP)	0.758 ± 0.018	0.618 ± 0.008	0.390 ± 0.007	0.507 ± 0.009
Dense(MO-RDF)	0.571 ± 0.023	0.659 ± 0.037	0.345 ± 0.026	0.468 ± 0.024
Dense(MFP, MO-RDF)	0.824 ± 0.015	0.747 ± 0.006	0.328 ± 0.006	0.427 ± 0.008
**Dense(E_{ZINDO}, MO-RDF)**	$\mathbf{0.900 \pm 0.010}$	$\mathbf{0.920 \pm 0.004}$	$\mathbf{0.169 \pm 0.006}$	$\mathbf{0.238 \pm 0.005}$
Dense(E_{ZINDO}, MFP)	0.877 ± 0.011	0.820 ± 0.005	0.278 ± 0.008	0.359 ± 0.010
Dense(E_{ZINDO}, MO-RDF, MFP)	0.897 ± 0.009	0.861 ± 0.003	0.243 ± 0.006	0.315 ± 0.007

B.4 Test set prediction plots

Selected prediction plots for several model variants applied to the 9500 test set, as shown in Fig. S7. (a) and (b) show the best two performing models that have nearly identical distributions. (c) and (d) are the stand-alone AttentiveFP models with default atom (DA) or electronic atom (EA) descriptors. (e) and (f) show the best performing dense-only networks that used the ZINDO energy, MO-RDF, and MorganFP descriptor sets. Note the r, MAE and RMSE here are different from the results tables, as these metrics were obtain by comparing averaged predictions against the true data. This is achieved by averaging each individual data point across all 10 fold models, giving y_{pred}^{avg}, which is a more reliable estimate. The metrics are determined between y_{pred}^{avg} and y_{true}, rather than comparing individual folds y_{pred}^{fold} in turn and averaging the 10 metrics after.

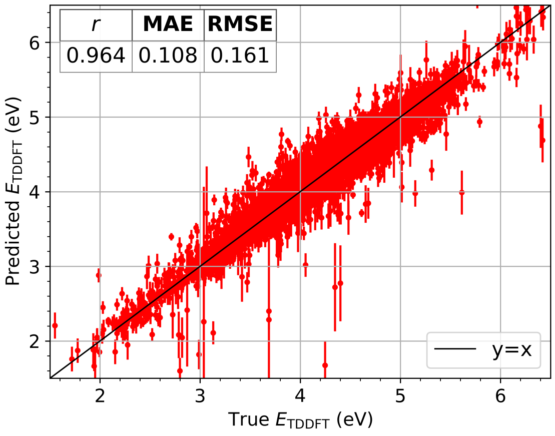

(a) Dense(MPNN(DA, EA), E_{ZINDO})

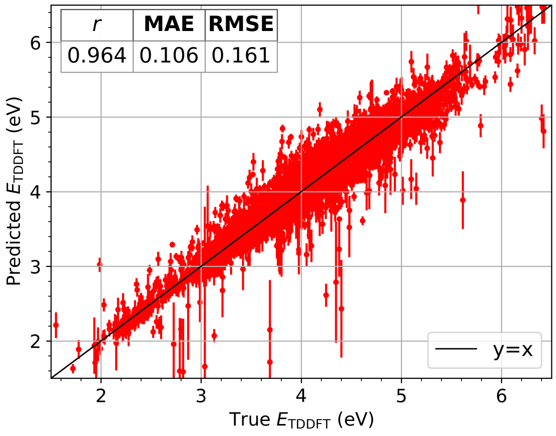

(b) Dense(MPNN(DA, EA), E_{ZINDO}, MO-RDF)

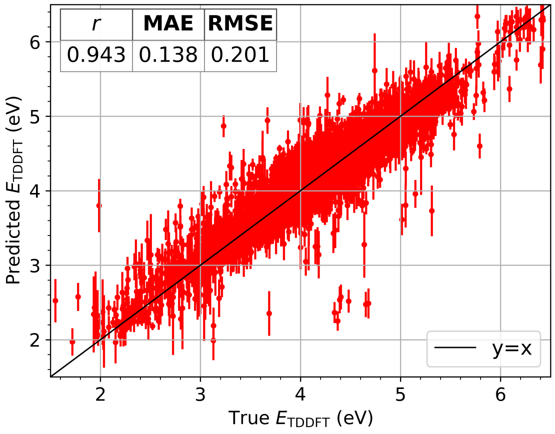

(c) MPNN(DA)

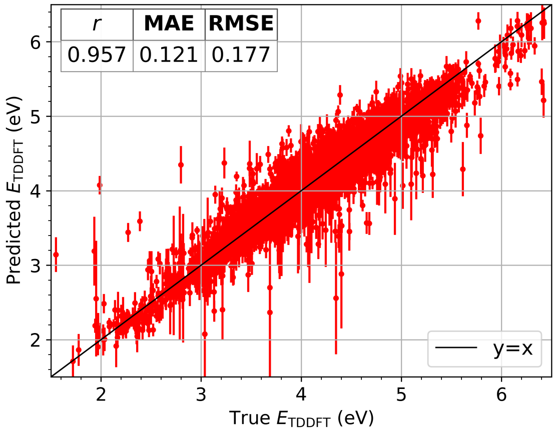

(d) MPNN(DA, EA)

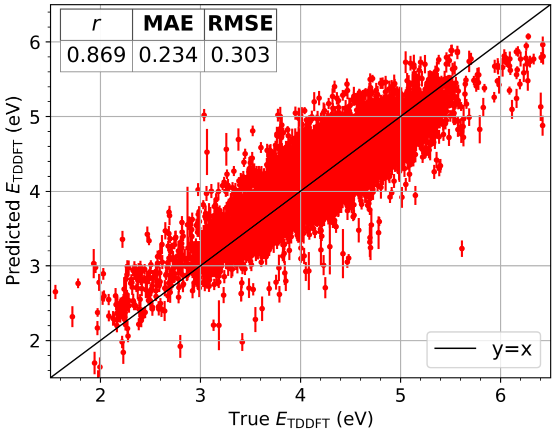

(e) Dense(E_{ZINDO}, MO-RDF, MFP)

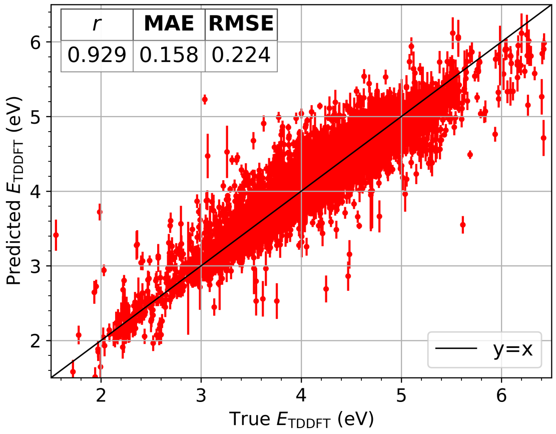

(f) Dense(E_{ZINDO}, MO-RDF)

Figure S7: Test set predictions for different model variants

C Comparisons between experiment and TDDFT

C.1 Evaluation of various TDDFT methods to experimental values

An analysis by Xia Wu et. al. [15] evaluated experimental values of excitation energies, oscillator strength and reorganisation energy to a range of calculation methods (B3LYP, M06-2X, wB97XD / 6-31G(d), def2-SVP, def2-TZVP) with and without solvation effects for a dataset of 71 dyes. Table S3 shows metrics between experimental values and different calculation methods for the S_1 energies of a 71 molecule dataset from this work [15], which highlights how TDDFT has a strong relationship to experiment, with a correlation of 0.95 when averaged across a range of functionals and basis sets. Note, as discussed in the work by Wu et. al, solvation effects are incredibly important when comparing DFT to experimental values. However, in the context and scope of this work it is sufficient to quote the results without solvation effects.

Table S3: Metrics for experimental data versus theoretical TDDFT calculations using 67 molecule data from work by Xia Wu et. al. in ref. [15]. Correlations comparing various methods (without solvation models) to experimental S_1 values range from 0.936 to 0.954. The final row shows the column averages across all methods.

**Method**	$\mathbf{R^2}$	**r**	**RMSE (eV)**	**MAE (eV)**
B3LYP/6-31g(d)	0.885	0.941	0.359	0.313
B3LYP/def2-SVP	0.876	0.936	0.355	0.308
B3LYP/def2-TZVP	0.884	0.940	0.320	0.273
M06-2X/6-31g(d)	0.905	0.952	0.557	0.511
M06-2X/def2-SVP	0.900	0.949	0.544	0.498
M06-2X/def2-TZVP	0.905	0.951	0.493	0.447
ωB97XD/6-31g(d)	0.910	0.954	0.585	0.544
ωB97XD/def2-SVP	0.905	0.951	0.577	0.535
ωB97XD/def2-TZVP	0.911	0.954	0.515	0.472
Average	0.898	0.948	0.478	0.433

C.2 Evaluation of M06-2X/3-21G* TDDFT against experiment and M06-2X/def2-TZVP

A set of 100 molecules with experimental E_{S_1} values were taken from the Handbook of Photochemistry [16] and calculation data obtained for these at the (M06-2X/3-21G* // BLYP35/3-21G*) and (M06-2X/def2-TZVP // BLYP35/def2-TZVP) levels, as per the work by Padula et. al. in ref. [17]. In Fig S8, we see in (a) S_1 energy comparisons of the low-level (M06-2X/3-21G* // BLYP35/3-21G*) method against experiment, with a correlation of 0.954 and in (b) the low-level 3-21G* against the higher level def2-TZVP basis set, with a correlation of 0.985. This validates our use of the less accurate 3-21G* basis set, which offers a 90% reduction in calculation time against the more accurate but slower def2-TZVP.

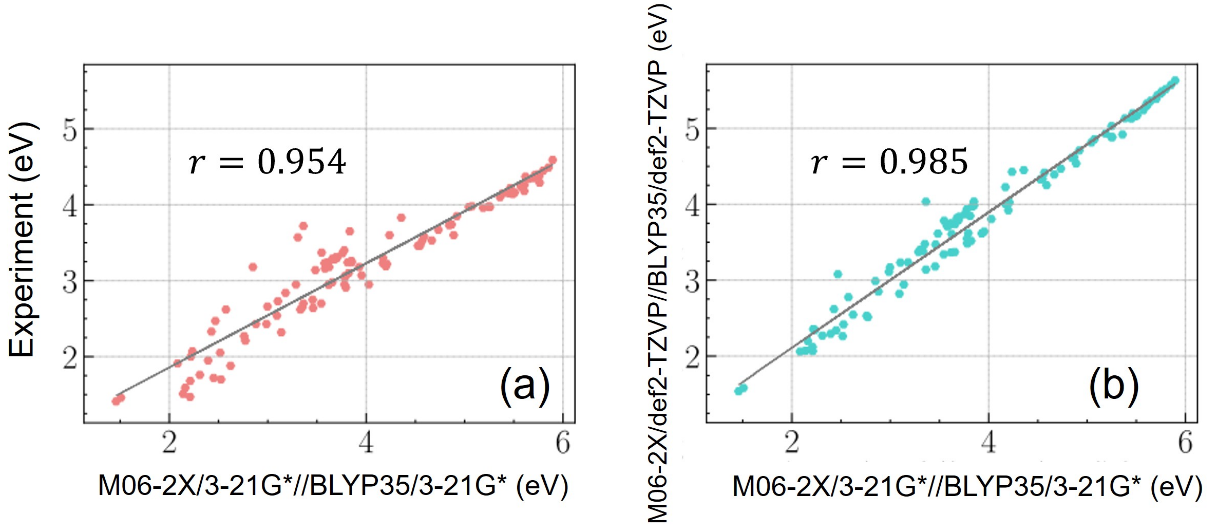

Figure S8: Comparison of S_1 energies between low-level (BLYP35/3-21G* // M06-2X/3-21G*) TDDFT predictions to (a) experimental values and (b) high-level (BLYP35/def2-TZVP // M06-2X/def2-TZVP) predictions.

C.3 Analysis of 500 HAC sample across several levels of theory

As a final evaluation of the model, the 9500 test set was sorted in ascending order by heavy atom count (HAC) and every 19th molecule selected to form a reproducible 500 molecule HAC-sorted sample. This resampling was done to avoid the cost of recomputing all 9500 molecules at the def2-TZVP levels and due to the fact that 500 points are statistically sufficient to evaluate the distributions. For this HAC sample, using the same B35LYP/3-21G* optimised geometries to ensure conformational consistency, TDDFT single point calculations were performed at the M06-2X/def2-SVP and M06-2X/def2-TZVP levels so their S_1 energies could be compared to ZINDO and ΔML-ZINDO model predictions. The correlations between the methods are reported in Table S4. The distribution of calculation time and molecule counts are shown in Fig S9. We see that ZINDO calculations are all under 20 seconds (and by association, ΔML-ZINDO), while for even the least expensive TDDFT method, the minimum calculation time at 10 heavy atoms is 2 minutes and maximum at 35 heavy atoms is 2 hours.

Table S4: Correlations for S_1 energies for a 500-HAC sample at various calculation method pairs, which in order of increasing accuracy are: ZINDO, ΔML-ZINDO, 3-21G*, def2-SVP, and def2-TZVP. Note, all TDDFT methods used the M06-2X functional, which has been dropped from the notation for brevity.

**Method X**	**Method Y**	**r**
def2-SVP	def2-TZVP	0.993
3-21G*	def2-TZVP	0.973
ΔML-ZINDO	def2-TZVP	0.942
ZINDO	def2-TZVP	0.758
ΔML-ZINDO	3-21G*	0.969
ZINDO	3-21G*	0.765

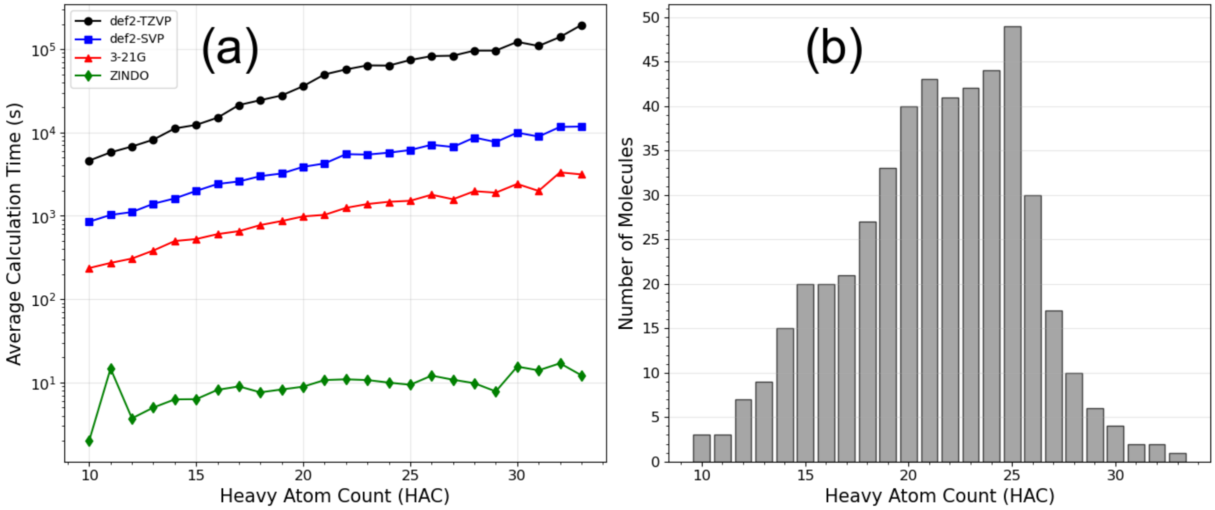

Figure S9: Distributions of (a) calculation time and (b) molecule count with heavy atom count for a 500-HAC sample at various levels of calculation: ZINDO, 3-21G*, def2-SVP, and def2-TZVP. Note, the shape of histogram for the 500 HAC sample is the similar to the full 9500 test set.

Plots of the energy comparisons are shown in Fig S10, where (a) shows excellent correlation between our original target level of theory (3-21G*) and the considerably more accurate (def2-TZVP) basis set. (c) and (d) show the ΔML-ZINDO model against def2-TZVP and 3-21G*, respectively. Similarly (e) and (f) show this for ZINDO data without ML corrections. Although we do not have experimental values available for the 500-HAC sample, since 3-21G* and def2-TZVP have a good correlation to experimental values by approximately 0.95 (Fig S8), we can claim that the ΔML-ZINDO model can approximate experimental S_1 energies sufficiently for use of this model in screening efforts. For example, for up to 10,000 molecules DFT calculations are viable, but for up to 100,000 or more, a ΔML-ZINDO can be applied as a pre-DFT screening step. Moreover, as our best models achieved similar correlations as TDDFT to experiment, any further improvements in accuracy at modelling the TDDFT values will not have a significant impact upon virtual screening efforts.

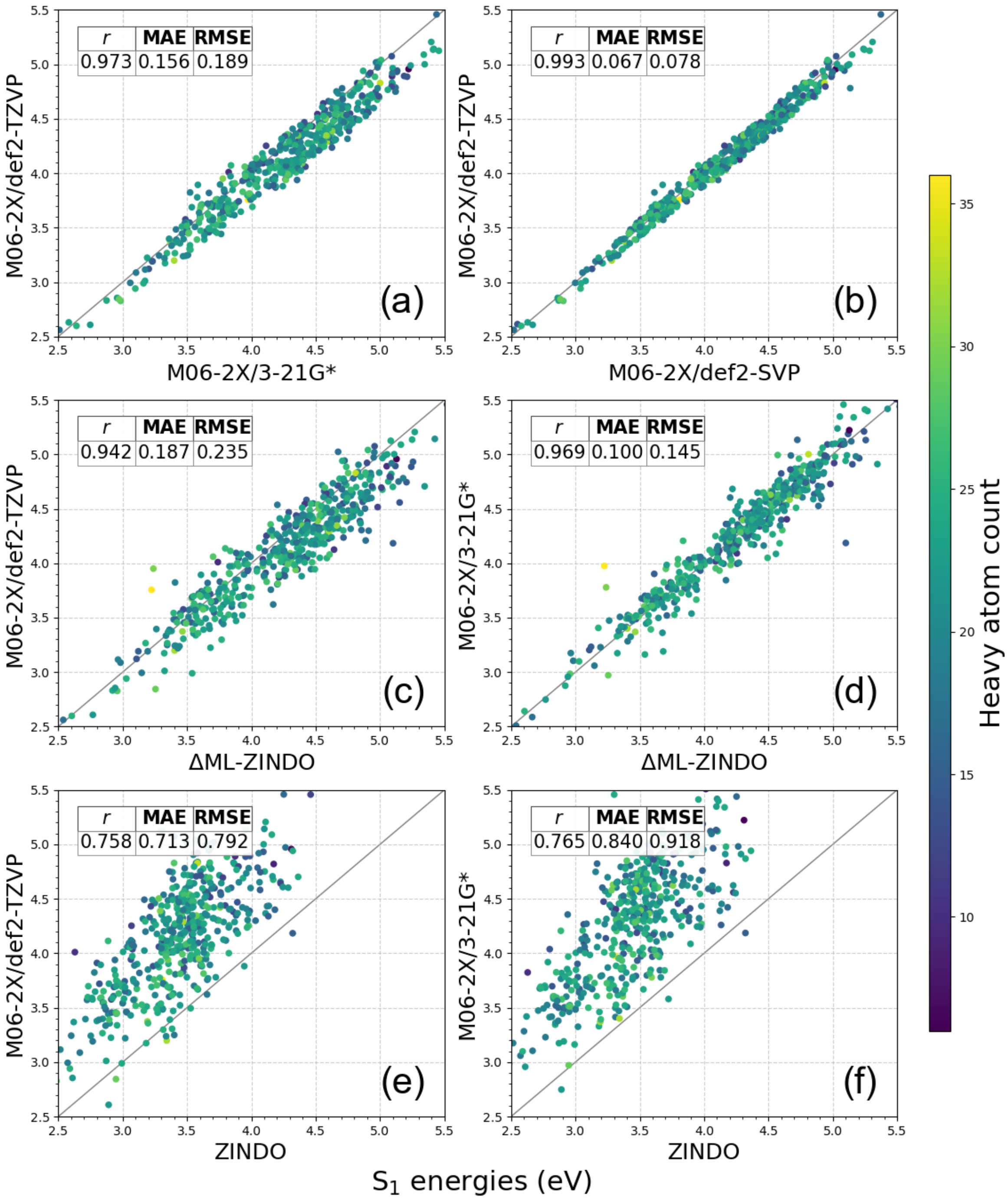

Figure S10: S_1 energy comparison for a 500-HAC sample at various levels of calculation, which in order of increasing accuracy are: ZINDO, ΔML-ZINDO, 3-21G*, def2-SVP, and def2-TZVP.

D Adapting ΔML-ZINDO for oscillator strength prediction

It is useful to explore whether the proposed methodology can be employed to discover emissive molecules and therefore estimate the oscillator strength of the excited states. In general, machine learning oscillator strength can be a difficult endeavour, but is of high importance in excited states modelling [18–21]. To adapt the model for oscillator strength (f) prediction, no changes were made to the framework except for the swapping of the S_1 E_{ZINDO} and E_{TDDFT} terms for their oscillator strength equivalents, f_{ZINDO} and f_{TDDFT}. The Dense(MPNN(DA, EA), f_{ZINDO}, MO-RDF) model was trained via the same 10-fold ensemble process as before and tested on the S_1 oscillator strengths from the 9500 test set. On the validation set, the oscillator strength for ZINDO to M06-2X/3-21G* TDDFT gave a correlation of 0.579, while ΔML-ZINDO to TDDFT improved the correlation by 35% to 0.779. For the test set, ZINDO to TDDFT gave a correlation of 0.524, while the ΔML-ZINDO model improved the correlation by 60% to 0.839, as shown in Fig S11. The method is particularly effective at identifying emissive molecules, where 70% of molecules predicted to have $f > 0.4$ by TDDFT were correctly predicted by our ΔML-ZINDO model. While not the main focus of this work, this shows how our ΔML framework can be adapted for other electronic properties.

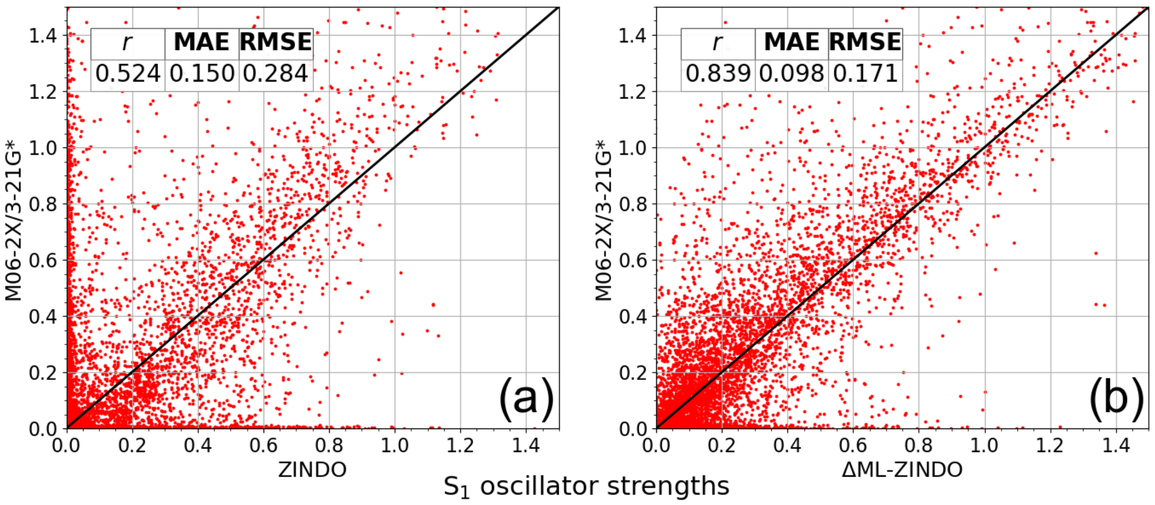

Figure S11: Test set plots for ZINDO (a) and ΔML-ZINDO (b) predictions against TDDFT for S_1 state oscillator strengths. This data includes states incorrectly inverted by 3-21G* in reference to def2-TZVP singlet excitation state ordering.

One issue identified in this work was the different ordering of bright and dark excited states between different levels of theory, which complicates the correction of systematic errors. Fig. S12, (a) shows def2-TZVP versus 3-21G* level oscillator strengths for the 500-HAC sample, where we clearly see a vertical line of points where 3-21G* predicts near-zero oscillator strengths in contrast to the larger values predicted by def2-TZVP. This occurs due to near-degeneracy between the low-lying singlet excited states which are ordered differently in the two calculations. As the larger def2-TZVP basis set is generally accepted as more accurate, we can work under the assumption that the def2-TZVP singlet state ordering is the correct order (ignoring any systematic biases of the def2-TZVP set). Then we can use def2-TZVP as a reference to determine which 3-21G* states are inverted and remove these degenerate states from the distribution (i.e. which states have near-zero S_1 f at 3-21G* level but much larger def2-TZVP f values), as shown in (b). However, when training an

ML screening tool, one would only have access to the 3-21G* data and consequently there is a need for a metric to quantify degeneracy without calculating the def2-TZVP reference.

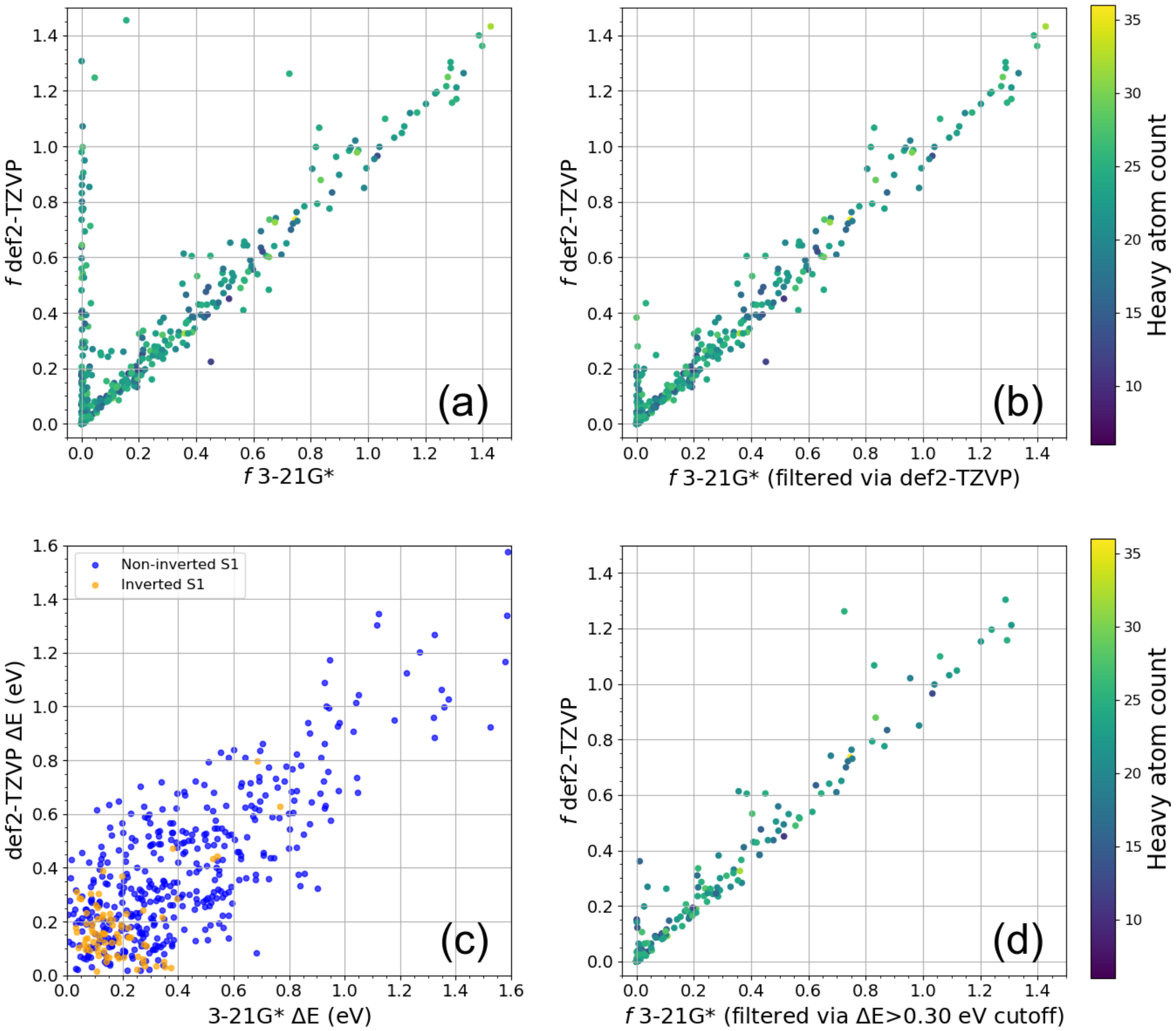

Figure S12: Analysis of singlet state inversion between 3-21G* and def2-TZVP for the 500-HAC sample. (a) shows a vertical of line where 3-21G* incorrectly inverts states compared to the more accurate def2-TZVP basis set. These can be removed via comparison to def2-TZVP (b) or by removing potentially degenerate states (c) via a 0.3 eV cutoff (d).

Degenerate states typically occur when the energy differences between the first and second excited state ($\Delta E = E_{S_2} - E_{S_1}$) are small, which can be seen in (c) in orange where the majority of molecules with 'incorrectly' inverted states with respect to an idealised def2-TZVP reference lie below 0.30 eV. In (d), after removing any molecules whose 3-21G* ΔE are below this cutoff, the vertical line of incorrectly inverted states is no longer present (albeit at the loss of many correctly predicted states).

We applied the $\Delta E < 0.30$ cutoff to our training set, which reduced it from 7602 molecules to 4031 and similarly for the testing data, which went from 9500 to 4996. With this filtered dataset

we retrained our ΔML model. On the validation set the ZINDO to TDDFT/3-21G* correlation was 0.749, while the ΔML-ZINDO model gave a correlation of 0.894, For the test set, the ZINDO to TDDFT correlation was 0.693 and the ΔML-ZINDO to TDDFT correlation was 0.910, an improvement by 31%, as shown in Fig. S13. These results highlight how, after accounting for the known issue of basis set dependent inversion of states, the ΔML-ZINDO framework can easily be adapted for oscillator strength prediction. While the ΔE cutoff is appropriate for these demonstration purposes, future work in oscillator strength should identify a more robust metric for the identification of incorrectly inverted states. Alternatively, to avoid the limitations of the 3-21G* basis set for applications where oscillator strength prediction is important, the cost of generating low- and high-level training data with more accurate methods may be justified (i.e provide a ΔML mapping from TDDFT/3-21G* to TDDFT/def2-TZVP).

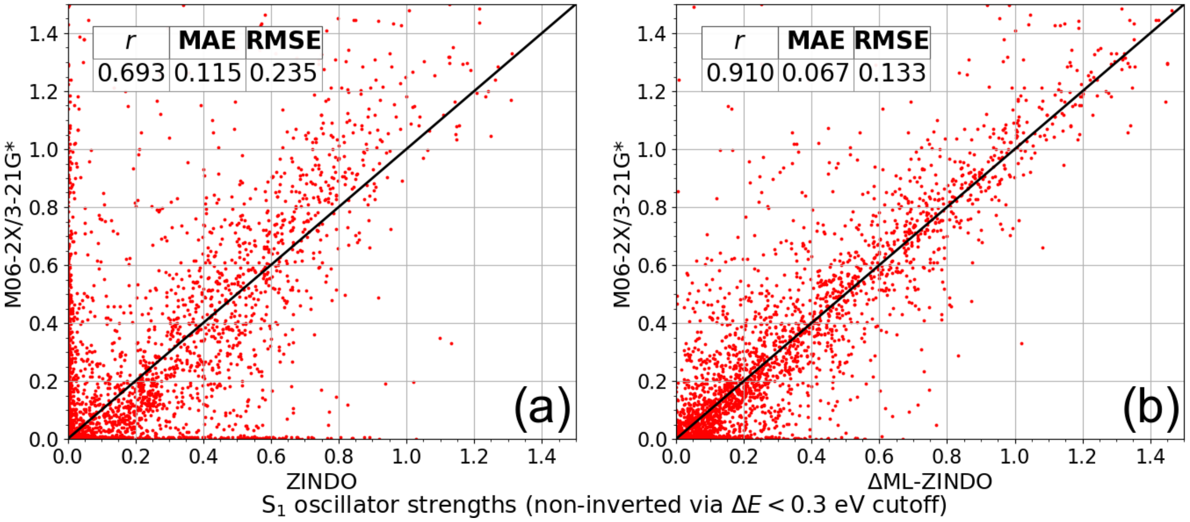

Figure S13: Test set plots for (a) ZINDO and (b) ΔML-ZINDO to TDDFT S_1 state oscillator strengths after removal of inverted states using $\Delta E < 0.3$ eV as a cutoff metric.

References

(1) Omar, Ö. H.; Xie, X.; Troisi, A.; Padula, D. Identification of Unknown Inverted Singlet–Triplet Cores by High-Throughput Virtual Screening. *Journal of the American Chemical Society* **2023**, *145*, 19790–19799, DOI: 10.1021/jacs.3c05452.

(2) Sterling, T.; Irwin, J. J. ZINC 15 – Ligand Discovery for Everyone. *J. Chem. Inf. Model* **2015**, *55*, 2324–2337, DOI: 10.1021/acs.jcim.5b00559.

(3) Weininger, D. SMILES, a Chemical Language and Information System. 1. Introduction to Methodology and Encoding Rules. *Journal of Chemical Information and Computer Sciences* **1988**, *28*, 31–36, DOI: 10.1021/ci00057a005.

(4) Fushiki, T. Estimation of Prediction Error by Using K-Fold Cross-Validation. *Statistics and Computing* **2011**, *21*, 137–146, DOI: 10.1007/s11222-009-9153-8.

(5) Oz, M. A Method for Measuring Similarity or Distance of Molecular and Arbitrary Graphs Based on a Collection of Topological Indices. *Journal of Chemometrics* **2025**, *39*, e70047, DOI: 10.1002/cem.70047.

(6) Carhart, R. E.; Smith, D. H.; Venkataraghavan, R. Atom Pairs as Molecular Features in Structure–Activity Studies: Definition and Applications. *J. Chem. Inf. Comput. Sci.* **1985**, *25*, 64–73, DOI: 10.1021/ci00046a002.

(7) Awale, M.; Reymond, J.-L. Atom Pair 2D-Fingerprints Perceive 3D-Molecular Shape and Pharmacophores for Very Fast Virtual Screening of ZINC and GDB-17. *Journal of Chemical Information and Modeling* **2014**, *54*, 1892–1907, DOI: 10.1021/ci500232g.

(8) Reddy, S. K.; Reddy, S. V. G. In *AIP Conference Proceedings*, AIP Publishing: 2025; Vol. 3325, p 030038, DOI: 10.1063/5.0291801.

(9) Duke, R.; Yang, C.-H.; Ganapathysubramanian, B.; Risko, C. Evaluating Molecular Similarity Measures: Do Similarity Measures Reflect Electronic Structure Properties? *Journal of Chemical Information and Modeling* **2025**, *65*, 4311–4319, DOI: 10.1021/acs.jcim.5c00175.

(10) Cuevas-Zuviría, B.; Pacios, L. F. Analytical model of electron density and its machine learning inference. *J. Chem. Inf. Model* **2020**, *60*, 3831–3842, DOI: 10.1021/acs.jcim.0c00197.

(11) Politzer, P.; Murray, J. S. Atoms do exist in molecules: analysis using electrostatic potentials at nuclei. *Mol. Phys.* **2022**, DOI: 10.1080/00268976.2022.2101563.

(12) Buzelli, T.; Ipaves, B.; Gollino, F.; Almeida, W. P.; Galvão, D. S.; da Silva Autreto, P. A. Machine learning-based analysis of electronic properties as predictors of anticholinesterase activity in chalcone derivatives. *Comput. Theor. Chem.* **2025**, 115268, DOI: 10.1016/j.comptc.2025.115268.

(13) Gromov, O. I. Neural Mulliken Analysis: Molecular Graphs from Density Matrices for QSPR on Raw Quantum-Chemical Data. *J. Chem. Theory Comput.* **2024**, DOI: 10.1021/acs.jctc.5c00425.

(14) Jensen, F., *Introduction to computational chemistry*; John wiley & sons: 2017.

(15) Wu, X.; Xie, X.; Troisi, A. Calibration of several first excited state properties for organic molecules through systematic comparison of TDDFT with experimental spectra. *J. Mater. Chem. C* **2024**, *12*, 18886–18892, DOI: 10.1039/D4TC03511A.

(16) Montalti, M.; Credi, A.; Prodi, L.; Gandolfi, M. T., *Handbook of Photochemistry*; CRC Press: 2006, DOI: 10.1201/9781420015195.

(17) Padula, D.; Omar, Ö. H.; Nematiaram, T.; Troisi, A. Singlet fission molecules among known compounds: finding a few needles in a haystack. *Energy Environ. Sci* **2019**, *12*, 2412–2416, DOI: 10.1039/C9EE01508F.

(18) Kang, B.; Seok, C.; Lee, J. Prediction of Molecular Electronic Transitions Using Random Forests. *Journal of Chemical Information and Modeling* **2020**, *60*, 5984–5994, DOI: 10.1021/acs.jcim.0c00698.

(19) Westermayr, J.; Marquetand, P. Machine Learning for Electronically Excited States of Molecules. *Chemical Reviews* **2021**, *121*, 9873–9926, DOI: 10.1021/acs.chemrev.0c00749.

(20) Katubi, K. M.; Alsaiari, N. S.; Naeem, S.; Al-Buriahi, M. Machine Learning Assisted Designing of Dyes and Oscillator Strength Prediction: Chemical Space Generation and Visualization. *ChemistrySelect* **2025**, *10*, e202500223, DOI: 10.1002/slct.202500223.

(21) Sanyam; Das, B.; Mondal, A. Physics-Guided Machine Learning of Excited-State Properties for the Design of High-Performance TADF Emitters. *Journal of Materials Chemistry A* **2025**, *13*, 33553–33570, DOI: 10.1039/D5TA03374H.
